# Meta-analysis of gene expression disease signatures in colonic biopsy tissue from patients with ulcerative colitis

**DOI:** 10.1038/s41598-021-97366-5

**Published:** 2021-09-14

**Authors:** Bryan Linggi, Vipul Jairath, Guangyong Zou, Lisa M. Shackelton, Dermot P. B. McGovern, Azucena Salas, Bram Verstockt, Mark S. Silverberg, Shadi Nayeri, Brian G. Feagan, Niels Vande Casteele

**Affiliations:** 1 Alimentiv, Inc, 100 Dundas St, Suite 200, London, ON N6A 5B6 Canada; 2 Department of Medicine, University of Western Ontario, London, ON Canada; 3 Department of Epidemiology and Biostatistics, University of Western Ontario, London, ON Canada; 4 F. Widjaja Foundation Inflammatory Bowel and Immunobiology Research Institute, Cedars-Sinai Medical Center, 8700 Beverly Blvd, Los Angeles, CA 90048 USA; 5 Institut d’Investigacions Biomèdiques August Pi i Sunyer (IDIBAPS), Centro de Investigación Biomédica en Red de Enfermedades Hepáticas y Digestivas (CIBERehd), Casanova, 143 08036 Barcelona, Spain; 6 Department of Gastroenterology and Hepatology, University Hospitals Leuven, KU Leuven, Oude Markt 13, 3000 Leuven, Belgium; 7 Department of Chronic Diseases, Metabolism and Ageing, Translational Research in Gastrointestinal Disorders (TARGID) - IBD Unit, KU Leuven, Leuven, Belgium; 8 Mount Sinai Hospital Inflammatory Bowel Disease Centre, University of Toronto, 600 University Ave, Toronto, ON M5G 1X5 Canada; 9 Zane Cohen Centre for Digestive Diseases, Lunenfeld-Tanenbaum Research Institute, Sinai Health System, 600 University Ave, Toronto, ON M5G 1X5 Canada; 10 Division of Gastroenterology, Department of Medicine, University of California San Diego, IBD Center, 9500 Gilman Drive #0956, La Jolla, CA 92093 USA

**Keywords:** Ulcerative colitis, Transcriptomics, Microarrays

## Abstract

Publicly available ulcerative colitis (UC) gene expression datasets from observational studies and clinical trials include inherently heterogeneous disease characteristics and methodology. We used meta-analysis to identify a robust UC gene signature from inflamed biopsies. Eight gene expression datasets derived from biopsy tissue samples from noninflammatory bowel disease (IBD) controls and areas of active inflammation from patients with UC were publicly available. Expression- and meta-data were downloaded with GEOquery. Differentially expressed genes (DEG) in individual datasets were defined as those with fold change > 1.5 and a Benjamini–Hochberg adjusted *P* value < .05. Meta-analysis of all DEG used a random effects model. Reactome pathway enrichment analysis was conducted. Meta-analysis identified 946 up- and 543 down-regulated genes in patients with UC compared to non-IBD controls (1.2 and 1.7 times fewer up- and down-regulated genes than the median of the individual datasets). Top-ranked up- and down-regulated DEG were *LCN2* and *AQP8*. Multiple immune-related pathways (e.g., ‘Chemokine receptors bind chemokine’ and ‘Interleukin-10 signaling’) were significantly up-regulated in UC, while ‘Biological oxidations’ and ‘Fatty acid metabolism’ were downregulated. A web-based data-mining tool with the meta-analysis results was made available (https://premedibd.com/genes.html). A UC inflamed biopsy disease gene signature was derived. This signature may be an unbiased reference for comparison and improve the efficiency of UC biomarker studies by increasing confidence for identification of disease-related genes and pathways.

## Introduction

Ulcerative colitis (UC) is a chronic relapsing–remitting disease of the large intestine that is associated with both genetic and environmental risk factors. Ulcerative colitis is characterized by inflammation of the mucosa and submucosa, a loss of epithelial barrier integrity, and dysregulated immune responses. Medical management of patients with moderately to severely active UC includes therapies targeting key aspects of the inflammatory cascade, such as pro-inflammatory cytokines (tumor necrosis factor [TNF] antagonists and interleukin [IL]-12/23 antibodies), signaling proteins (e.g., Janus kinase family inhibitors), or immune cell trafficking (vedolizumab)^1^. Despite the range of available therapies, many UC patients experience primary non-response or secondary loss of response to treatment and must cycle through several therapies to achieve remission; this process is currently based upon trial and error. Thus, more accurate identification of disease subtypes that would benefit from targeted treatment with specific therapies is an area of active investigation and a large unmet need.

The clinical and therapeutic value of molecular (DNA, RNA, protein) subtyping of disease has been most notable in oncology^2^. Global transcriptome (RNA) analysis has been a particularly powerful and unbiased tool to aid in understanding of disease etiology, pathology, diagnosis, and subtypes, as well as for the identification of predictive markers of drug efficacy, molecular surrogates of disease activity, and/or pharmacodynamic markers^3^. Despite this abundant potential, the application of transcriptomics to clinical trial data has been frequently limited by small sample size, especially when subgroups of interest are considered (e.g., treatment responders versus non-responders). The high dimension-low sample size nature of the analysis^4^, in which the number of genes of interest far exceeds the number of available samples, is particularly problematic.

In UC, transcriptomics analysis of preclinical and clinical samples is frequently employed to aid in the understanding of disease pathogenesis, to discover new therapeutic targets, and to identify biomarkers^5^. The most appropriate samples available for these analyses are mucosal biopsies taken during sigmoidoscopy or colonoscopy, which are typically procured for histopathologic analysis of disease activity^6^, but also serve as source material for molecular analyses. Several aspects of tissue acquisition and processing may influence the results of transcriptomics analysis, including biopsy location within the colon and relationship to endoscopically visible active disease^7^.

Several publicly available transcriptomics datasets exist from patients with UC and healthy controls. Meta-analysis of data from multiple studies has been undertaken using various methodologies to explore the similarities between Crohn’s disease (CD) and UC^8,9^, to define disease signatures in peripheral blood mononuclear cells from patients with UC^10^, and to identify disease signatures associated with UC pathogenesis^11^.

We conducted random effects meta-analysis of publicly available UC microarray datasets derived from inflamed tissue biopsies to identify an overall list of UC disease signature genes, with a goal to improve the confidence for identification of disease-related genes from UC transcriptomic studies, and to explore aspects of study design that may lead to differences in gene expression.

## Methods

A search of the National Center for Biotechnology Information Gene Expression Omnibus (GEO)^12^ for microarray datasets using the terms [((ulcerative colitis) AND "Homo sapiens"[porgn] AND "gse"[Filter]) AND (("expression profiling by array"[DataSet Type]) AND "gse"[Filter]) AND ("gse"[Filter])] retrieved 85 datasets. Datasets were excluded for the following reasons: they did not contain both UC patients and non-inflammatory bowel disease (IBD) controls, they included only pediatric patients, there were fewer than 10 combined UC patients and non-IBD controls, samples were only taken from uninflamed mucosa or were included in other datasets, data was not expressed in intensity values or was z-score transformed, or samples were not processed at the same time. In 1 instance the original investigators were consulted to assess eligibility for inclusion of other datasets potentially containing samples in common with dataset GSE73611. These datasets were determined to contain the same samples and were therefore excluded. Eight datasets remained after exclusions (Supplementary Fig. S1). The subset of samples isolated from inflamed tissue of patients with active UC was selected from each dataset for this study. Additional methods are described in the Supplementary Methods.

## Results

### Datasets

A total of 8 microarray datasets deposited in the NCBI GEO database between 2009 and 2018 that were derived from intestinal tissue RNA from various cohorts and that included at least 10 patients with UC and non-IBD controls combined (see additional inclusion criteria in Supplementary Fig. S1), and which originated from a range of institutions and microarray platforms (Table 1) were identified, for a total of 251 samples from patients with UC and 94 samples from non-IBD controls.

**Table 1:** Microarray data series used for meta-analysis.

GEO series accession number	Sample source	PMID (publication year)	UC sample (N)	Non-IBD control samples (N)	Biopsy location	Sample storage method	Array platform	Disease duration	Disease activity measure	C reactive protein reported	Disease extent reported	Concomitant medications reported
13367	Yale University	19834973 (2010)	8	10	Descending colon	RNA*later*	Affymetrix human genome U133 plus 2.0 array	Years (> or < 10)	MCS	NR	NR	Yes
9452	University of Copenhagen	19177426 (2009)	8	5	Descending colon	RNA*later*	Affymetrix human genome U133 plus 2.0 array	Years of symptoms (patient level)	NR	NR	NR	Yes
53306	Johns Hopkins University	26034135 (2015)	12	12	Sigmoid colon or rectum	FFPE	Illumina humanHT-12 WG-DASL V4.0 R2 expression beadchip	Mean years	MCS and Matts	NR	Yes	Yes
38713	IDIBAPS	23135761 (2013)	15	13	Sigmoid colon or rectum	RNA*later*	Affymetrix human genome U133 plus 2.0 array	Mean years	MCS and Matts	NR	Yes	Yes
47908	Herlev Hospital	25358065 (2014)	39	15	Descending colon	RNA*later*	Affymetrix human genome U133 Plus 2.0 Array	Years (> or < 10)	MCS, MES	NR	Yes	Yes
73661	KU Leuven	27802155 (2017)	67	12	Edge of ulcer or most inflamed colonic segment	Snap frozen	Affymetrix human gene 1.0 ST array	Median years	MCS, MES, Geboes	NR	Yes	Yes
114527	CIC bioGUNE	30329026 (2018)	15	6	Rectum	NR	Illumina humanHT-12 WG-DASL V4.0 R2 expression beadchip	NR	MES	Yes	Yes	Yes
87466	Janssen R&D	29401083 (2018)	87	21	15–20 cm from anal verge	RNA*later*	Affymetrix HT HG-U133 + PM Array	Median years	MCS	Yes	Yes	Yes

*FFPE* formalin-fixed and paraffin-embedded, *GEO* Gene Expression Omnibus, *PMID* PubMed unique identifier, *IBD* inflammatory bowel disease, *MCS* Mayo Clinic Score, *MES* Mayo Endoscopic Score, *NR* not reported, *UC*, ulcerative colitis.

Data series meeting the inclusion criteria were identified and downloaded from Gene Expression Omnibus (GEO). Disease and study characteristics summarized in the table were extracted from publications (see PMID) that included samples used in the current study. In some cases, the samples used in the current study represented a subset of those utilized and/or described in the publications.

Datasets originated from the pharmaceutical industry (n = 1), academic hospitals or other European institutions (n = 5), and academic hospitals in the United States (n = 2). Two datasets included samples from clinical trials. Four datasets were run using the Affymetrix Human Genome U133 Plus 2.0 Array, 2 using Illumina HumanHT-12 WG-DASL V4.0 R2 expression beadchip, and 1 each using Affymetrix Human Gene 1.0 ST Array, and Affymetrix HT HG-U133 + PM Array.

The number of patients with UC and non-IBD controls in each dataset ranged from 8 to 87 (median 15) and 5 to 21 (median 12), respectively. Six datasets had more UC patients than non-IBD controls, including 4 with greater than two-times the number of UC patients than non-IBD controls.

The UC disease characteristics reported for the 8 datasets varied widely among the publications associated with the original study populations^13–20^. Seven of the 8 associated publications reported disease duration; 1 on the patient level, with the remaining 6 reporting summary statistics (median, mean, with interquartile range or standard deviation) or binary categorical (less than or greater than 10 years) data. Measurements used to define or describe endoscopic and/or histological disease activity for the original study populations included the total Mayo Clinic Score (MCS) or the endoscopic subscore of the MCS (n = 7), the Matt’s score (n = 2), and the Geboes score (n = 1). Measures of endoscopic or histological disease activity were not reported in 3 of the associated publications. C-reactive protein concentration was reported in 2, disease extent in 7, and concomitant medications in all publications.

Biopsy location and method for sample handling also varied considerably among the datasets. Biopsy samples from patients with UC were reported as originating from the descending colon in 3 datasets, the sigmoid colon or rectum in 2 datasets, the rectum in 1 dataset, the edge of an ulcer or the most inflamed colonic segment in 1 dataset, and 15 to 20 cm from the anal verge from locations representative of the degree of inflammation seen in the region in 1 dataset. Biopsy samples were reported as preserved in RNA*later* in 5 datasets, or snap frozen in liquid nitrogen, formalin-fixed and paraffin-embedded (FFPE), or the preservation method was not reported in 1 dataset each.

### Differentially expressed genes in individual datasets

**Figure 1:**
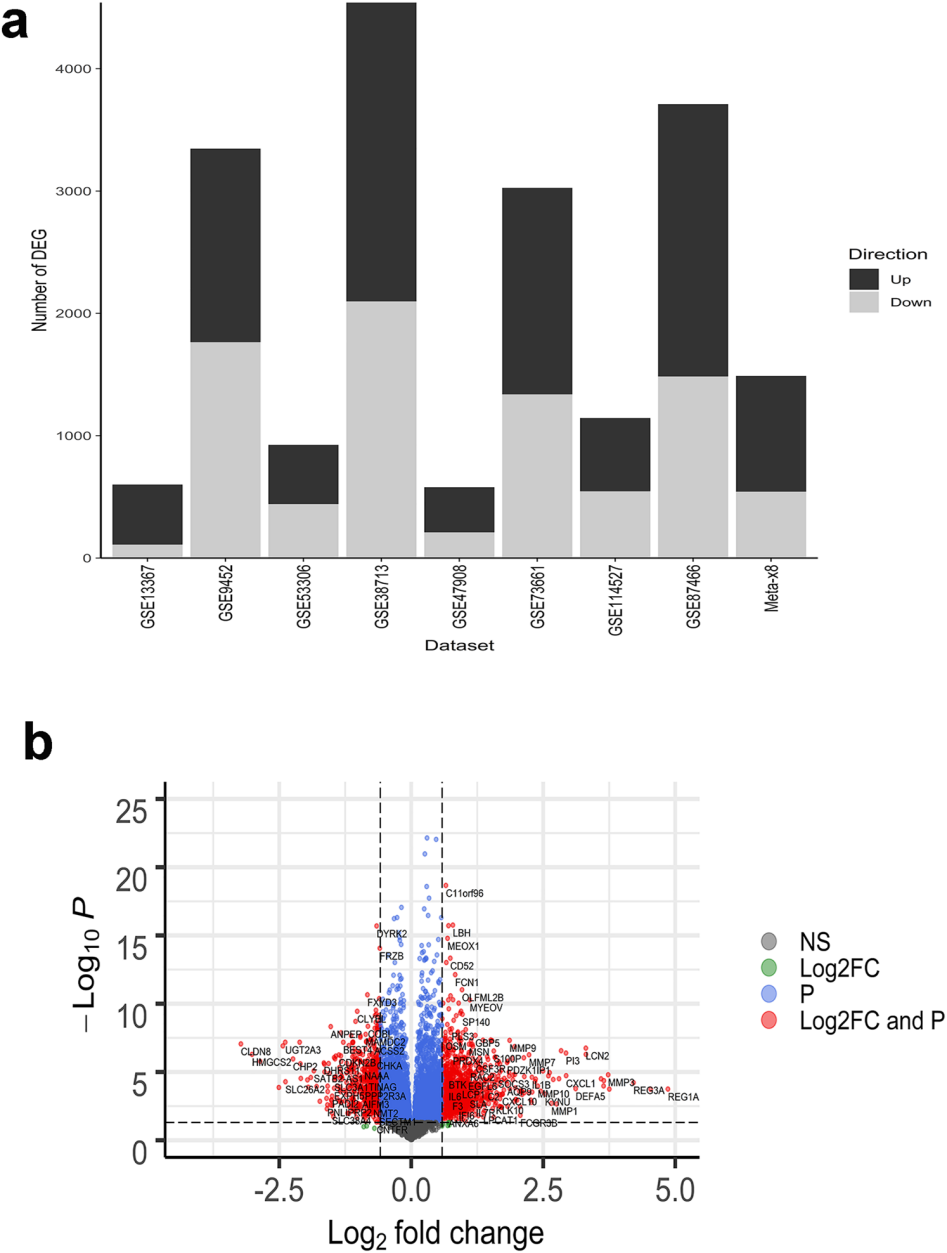
(**a**) Differentially expressed genes (up- or down-regulated log2-fold change [log2-FC] > 0.58 with adjusted *P* value < 0.05) for individual datasets and meta-analysis (Meta- × 8). Multiple Probesets for the same gene were summarized at Gene Symbol level (and counted as 1 differentially expressed gene). (**b**) Meta-analysis volcano plot of genes up- or down-regulated in ≥ 6 datasets. Dots to the left of 0 on the X-axis represent genes whose expression is lower in UC patients compared to healthy controls, whereas dots to right of 0 on X-axis represent genes whose expression is higher in patients with UC compared to healthy controls. Gray dots represent genes that do not meet the criteria for log2 fold change (FC) > 1.5 (up or down) or significant adjusted *P* value < 0.05. Green dots represent genes that meet the criterion for log2 FC > 1.5 (up or down) but not adjusted *P* value < 0.05. Blue dots represent genes with adjusted *P* value < 0.05 but not log2 FC > 1.5 (up or down). Red dots represent genes that meet both the log2 FC > 1.5 (up or down) and adjusted *P* value < 0.05 criteria. The horizontal dashed line is located at a value equivalent to the adjusted *P* value (0.05). Vertical lines are located at + and − 1.5 log2 FC.

Differentially expressed genes (DEG) in biopsies taken in areas of active inflammation from patients with UC and non-IBD controls were identified in each of the 8 datasets using identical methodology to allow direct comparison. The analysis was not adjusted for covariates such as age or sex since patient level data was not available for most datasets. In general, patients whose samples were included had undergone colonoscopy for UC disease surveillance, at screening for a clinical trial, or for suspicion of other gastrointestinal disorders. A wide range in the number of up- and down-regulated genes was observed. The median number of up-regulated genes was 1090 and ranged from 369 (GSE47908) to 2442 (GSE383713), while the median number of down-regulated genes was 942 and ranged from 110 (GSE13367) to 2098 (GSE38713) (Fig. 1). The DEG for each dataset are represented in individual volcano plots (Supplementary Figs. S2, S3, S4, S5, S6, S7, S8, S9).

### Meta-analysis

Given the wide range in the number of DEG for individual datasets, we conducted a random effects meta-analysis to identify similarities and differences in DEG across the 8 datasets. Compared to median values in the individual datasets, there were 1.2 times fewer up- and 1.7 fewer down-regulated DEG in the meta-analysis. The top 10 up- and down-regulated genes are shown in Table 2 and the complete list (meeting log2-fold change [FC > 0.58 [equivalent to 1.5-fold change], adjusted *P* value [< 0.05] criteria, and regulated in the same direction in 6 or more datasets) is provided in the Supplementary Dataset.

**Table 2:** Top 10 up- and down-regulated genes in meta-analysis. Genes were ranked using the TopConfects approach.

Symbol	Log2-FC (95% CI)	*P* value	Adjusted *P* value
**Up regulated**			
LCN2	3.31 (2.21, 4.41)	4.07E–09	1.80E–07
DUOXA2	3.31 (2.17, 4.45)	1.40E–08	5.16E–07
PI3	2.93 (1.93, 3.94)	1.08E–08	4.14E–07
CXCL8	2.84 (1.88, 3.8)	6.87E–09	2.79E–07
MMP3	3.73 (2.24, 5.22)	8.63E–07	1.60E–05
REG3A	4.21 (2.41, 6.01)	4.45E–06	6.07E–05
CHI3L1	3.6 (2.11, 5.08)	2.02E–06	3.21E–05
MMP7	2.23 (1.46, 3.01)	1.52E–08	5.54E–07
DUOX2	3.65 (2.12, 5.18)	2.91E–06	4.32E–05
MMP9	1.86 (1.27, 2.46)	8.78E–10	4.86E–08
**Down regulated**			
AQP8	− 4.27 (− 5.78, − 2.76)	3.02E–08	9.92E–07
CLDN8	− 3.23 (− 4.28, − 2.18)	1.77E–09	8.77E–08
HMGCS2	− 3.01 (− 4.06, − 1.97)	1.42E–08	5.21E–07
UGT2A3	− 2.39 (− 3.16, − 1.61)	1.30E–09	6.68E–08
GUCA2B	− 2.43 (− 3.23, − 1.63)	2.62E–09	1.23E–07
PCK1	− 2.86 (− 3.89, − 1.83)	5.69E–08	1.70E–06
TRPM6	− 2.11 (− 2.8, − 1.43)	1.29E–09	6.63E–08
CHP2	− 2.24 (− 3.03, − 1.44)	3.48E–08	1.11E–06
ANPEP	− 1.53 (− 1.98, − 1.07)	6.06E–11	4.81E–09
ADH1C	− 2.1 (− 2.87, − 1.33)	9.00E–08	2.52E–06

The adjusted *P* value was determined using the Benjamini Hochberg method.

Meta-analysis of DEG identified 8402 genes up- or down-regulated in the same direction in 6 or more datasets (Fig. 1). Of these, 946 up- and 543 down-regulated genes met the criteria for log2-fold change (FC > 0.58 [equivalent to 1.5-fold change]) and adjusted *P* value (< 0.05), 9 met the criterion for log2-FC, yet did not achieve significance at the adjusted *P* value, 5591 met the criterion for adjusted *P* value however not for log2-FC, and 1313 did not meet either criterion.

To evaluate the global DEG similarities between the individual datasets and the meta-analysis, we conducted a principal component analysis (PCA) to reduce data dimensions based on the log2-FC values. This analysis revealed 48.8% of the total variance in the first principal component (PC1) and 15.4% of the remaining variation in the second principal component (PC2). In PCA, the meta-analysis dataset was centered approximately between all datasets on the PC2 axis, confirming that the meta-analysis provided an effective ‘average’ representation of most of the individual datasets (Supplementary Fig. S10).

**Figure 2:**
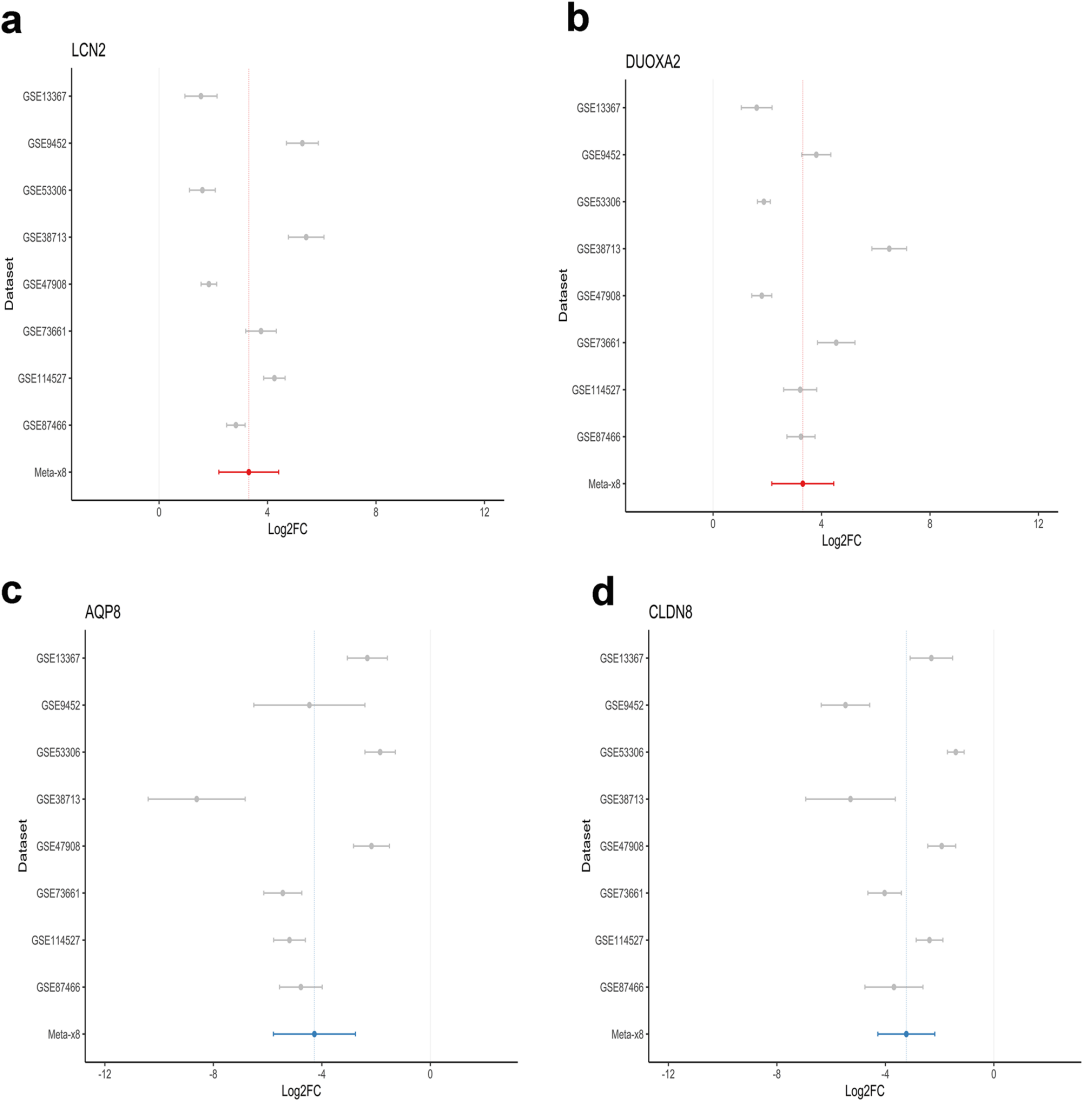
Forest plots of the mean log2 fold changes for the top 2 up-regulated genes identified in meta-analysis, *LCN2* (panel **a**) and *DUOXA2* (panel **b**). Data are shown for mean log2 fold changes for each gene in both the individual datasets and for the meta-analysis. The mean log2 fold change for each gene in the meta-analysis is represented by the vertical red dashed line, whereas the mean log2 fold change for the genes in each dataset is represented by grey dots. Whiskers for each mean value correspond to the 95% confidence interval. (**b**) Forest plots of the mean log2 fold changes for the top 2 down-regulated genes identified in meta-analysis, *AQP8* (panel **c**) and *CLDN8* (panel **d**). Data are shown for mean log2 fold changes for each gene in both the individual datasets and for the meta-analysis. The mean log2 fold change for each gene in the meta-analysis is represented by the vertical red dashed line, whereas the mean log2 fold change for the genes in each dataset is represented by grey dots. Whiskers for each mean value correspond to the 95% confidence interval.

To evaluate the influence of individual datasets on the results of the meta-analysis, forest plots were created that compared the mean log2-FC and confidence intervals for the top 2 up- (*LCN2* and *DUOXA2*, Fig. 2) and down- (*AQP8* and *CLDN8*, Fig. 2) regulated DEG. The mean log2-FC for *LCN2* in meta-analysis was 3.31 (95% CI 2.21, 4.41) (Fig. 2). *LCN2* was up-regulated in all 8 datasets, with a mean log2-FC ranging from 1.55 (GSE13367) to 5.43 (GSE38713). The 95% CIs for the mean changes were greater than 0 in all datasets. The overall expression pattern of *DUOXA2* was similar to *LCN2.* The mean log2-FC for *DUOXA2* in meta-analysis was 3.31 (95% CI 2.17, 4.45) (Fig. 2). *DUOXA2* was up-regulated in all 8 datasets with a mean log2-FC ranging from 1.61 (GSE13367) to 6.50 (GSE38713). The 95% CIs for the mean changes were greater than 0 in all datasets.

The mean log2-FC for *AQP8* in meta-analysis was − 4.27 (95% CI − 5.78, − 2.76) (Fig. 2). *AQP8* was down regulated in all 8 datasets with a mean log2-FC ranging from − 1.85 (GSE53306) to − 8.61 (GSE38713). The mean log2-FC for *CLDN8* in meta-analysis was − 3.23 (95% CI − 4.28, − 2.18) (Fig. 2) *CLDN8* was down-regulated in all 8 datasets, with a mean log2-FC ranging from − 1.40 (GSE53306) to − 5.47 (GSE9452).

### IBD susceptibility genes

To explore whether differential expression of any of the DEG identified in meta-analysis may be driven by known IBD susceptibility loci, we compared these genes to those previously identified in IBD genome-wide association^21^, expression quantitative trait loci^ (eQTL)22,23^, and methylation quantitative trait loci (mQTL)^24^ studies. Of the 241 IBD susceptibility genes identified by genome-wide association studies (GWAS), a total of 15 (6.2%) were also identified in meta-analysis (Table 3). Of 121 eQTL genes identified in 2 studies, 16 (13%) were also identified in meta-analysis (Table 3). Four mQTL^24^ were identified in the literature, none of which were included in the meta-analysis DEG. The overlap between DEG identified in meta-analysis and genes identified in IBD GWAS and eQTL suggests a possible mechanism for their regulation.

**Table 3:** Meta-analysis DEG previously identified as IBD susceptibility genes (either by GWAS or eQTL).

Gene	Meta-DEG log2-FC	Meta-DEG adj. *P*. val	Trait	Type
CXCL5	2.06	3.49E–03	UC	eQTL
NFKBIZ	1.13	9.68E–08	UC	GWAS
PTPRC	0.96	3.38E–03	IBD	GWAS
MUC1	0.93	2.41E–05	UC	eQTL
SLAMF8	0.87	8.49E–04	CD	GWAS
OSMR	0.85	2.88E–02	IBD	GWAS
PLCG2	0.82	1.25E–05	IBD	GWAS
CCR2	0.81	1.56E–02	CD	eQTL
NCF4	0.79	1.28E–04	CD	GWAS
GPR65	0.75	4.02E–04	IBD	eQTL
HLA-DQA1	0.75	1.44E–02	UC	eQTL
RSPO3	0.74	3.03E–04	CD	GWAS
RASGRP1	0.72	1.92E–04	IBD	GWAS
RASGRP1	0.72	1.92E–04	CD	eQTL
PRKCB	0.72	8.67E–06	IBD	GWAS
PRKCB	0.72	8.67E–06	UC	eQTL
MAP3K8	0.71	1.94E–16	IBD	GWAS
STAT4	0.65	1.42E–03	IBD	GWAS
ITGAL	0.65	1.54E–04	UC	eQTL
ITGAL	0.65	1.54E–04	UC	GWAS
STAT3	0.63	3.79E–06	CD	eQTL
IL18R1	0.61	2.79E–05	IBD	eQTL
CPEB4	0.61	1.47E–03	CD	eQTL
CPEB4	0.61	1.47E–03	IBD	GWAS
NR5A2	− 0.66	5.91E–10	IBD	GWAS
NXPE1	− 0.67	6.05E–04	UC	eQTL
SLC22A4	− 0.71	1.98E–04	IBD	eQTL
PGAP3	− 0.73	1.91E–05	IBD	eQTL
SLC22A23	− 0.79	5.84E–07	IBD	GWAS
NXPE4	− 1.27	2.31E–04	UC	eQTL
SLC22A5	− 1.43	2.17E–05	IBD	eQTL

Trait refers to the phenotype associated with single nucleotide polymorphism.

### Enrichment analysis

Gene set enrichment analysis was used to evaluate functions and processes potentially associated with the up- and down-regulated DEG in the individual datasets and to compare these pathways to those enriched in the DEG identified in meta-analysis.

**Figure 3:**
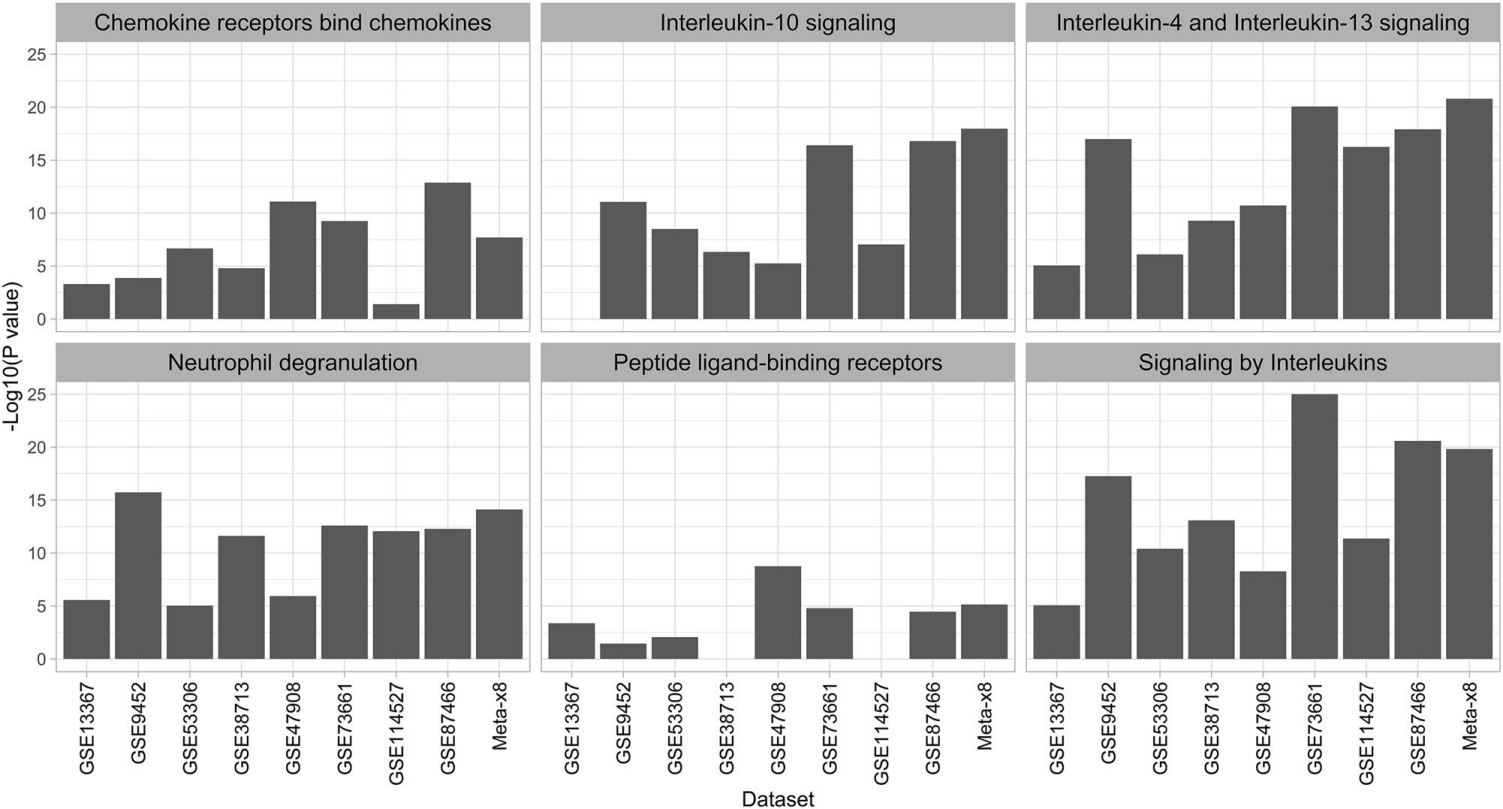
Enrichment of Immune-related Reactome pathways in individual datasets and meta-analysis (Meta- × 8) up-regulated genes. The − log10 of the adjusted *P* value is shown on the Y-axis with higher bars representing lower adjusted *P* values.

Ten Reactome pathways were enriched in the analysis of up-regulated DEG (Fig. 3 and Supplementary Fig. S11). Among these pathways, all 6 ‘Immune-related pathways’ in the Reactome database (Fig. 3; ‘Chemokine receptors bind chemokines’ [e.g., *CCL20* , *CCR7, CXCR2*], ‘Interleukin-10 signaling’ [e.g., *IL1R1*, *PTGS2*, *TIMP1*] ‘Interleukin-4 and Interleukin-13 signaling’ [e.g., *IL1A*, *IL6*, *MMP9*], ‘Neutrophil degranulation’ [e.g., *ALOX5*, *S100A8*, *SERPINA3*], ‘Peptide ligand-binding receptors’ [e.g., *ANXA1*, *C3*, *CXCL10*], and ‘Signaling by interleukins’ [e.g., *HGF*, *IL33*, *OSM*]) were enriched in the meta-analysis up-regulated DEG and in most of the individual dataset up-regulated DEG, except for ‘Interleukin-10 signaling’ in GSE13367 and ‘Peptide ligand-binding receptors’ in GSE38713 and GSE114527. All 4 ‘Extracellular matrix (ECM)-related pathways’ (Supplementary Fig. S11,‘Collagen degradation’ [e.g., *COL1A1*, *MMP1*, *MMP10*], ‘Degradation of extracellular matrix’ [e.g., *CD44*, *FBN1, LAMC1*], ‘ECM proteoglycans’ [e.g., *BGN, ITGA2*, *TNC*], and ‘Extracellular matrix organization’ [e.g., *ADAM9*, *CTSB*, *ICAM1*]) were also enriched in the meta-analysis up-regulated DEG and in the up-regulated DEG for all of the individual datasets except for ‘ECM proteoglycans’ and ‘Extracellular matrix organization’ in GSE53306.

**Figure 4:**
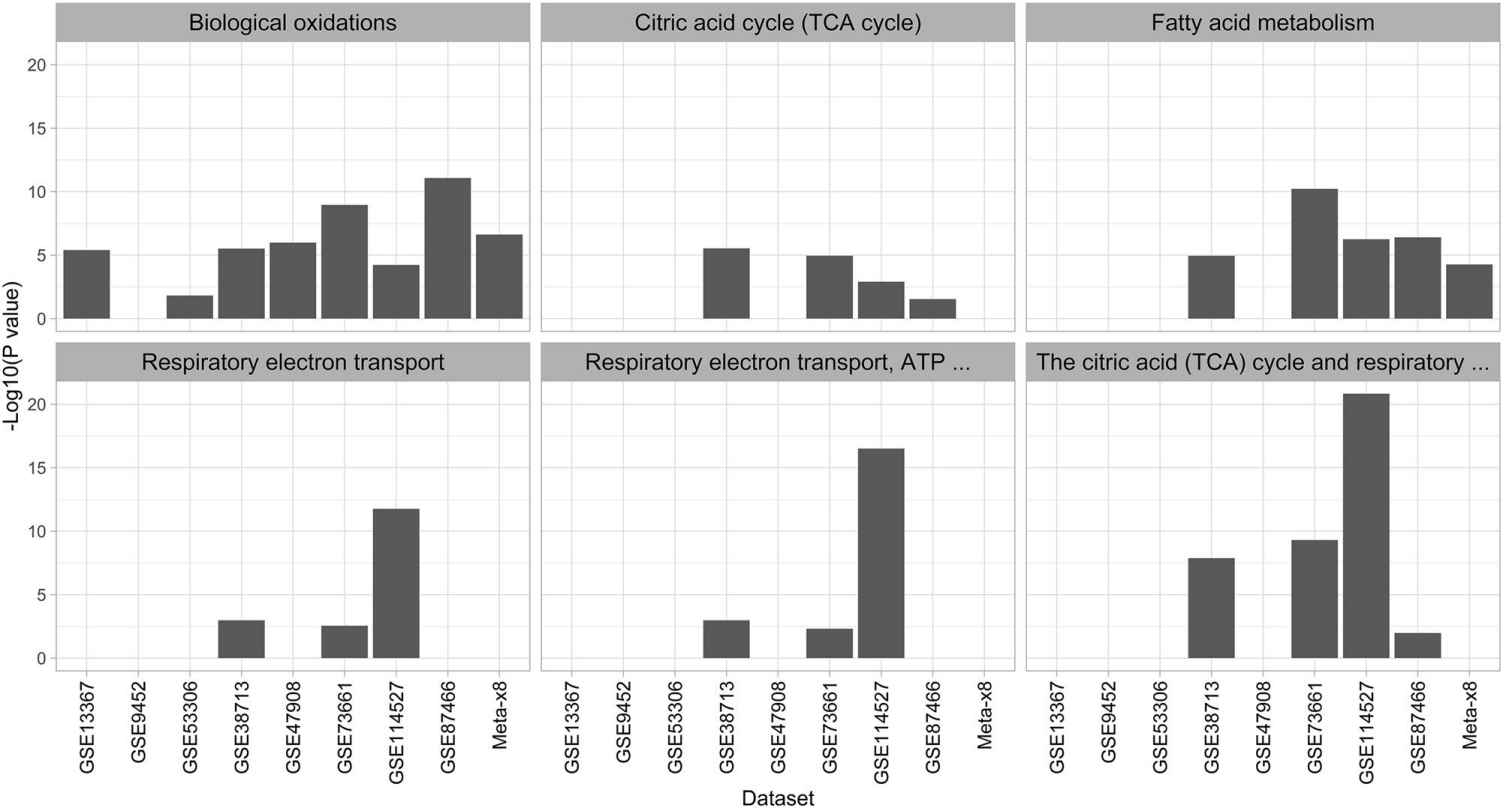
Enrichment of transport and modification-related Reactome pathways in individual dataset and meta-analysis (Meta- × 8) down-regulated genes. The − log10 of the adjusted *P* value is shown on the Y-axis with higher bars representing lower adjusted *P* values.

Eleven Reactome pathways were enriched in the analysis of down-regulated DEG (Fig. 4, and Supplementary Fig. S11). Large variation was observed in enrichment values within the 6 ‘Metabolism-related’ pathways amongst the datasets (Fig. 4). The meta-analysis down-regulated DEG were modestly enriched for ‘Biological oxidations’ (e.g., *CYP2J2*, *SLC26A2*, *UGT1A8*) and in 7 of the 8 individual dataset DEG, while ‘Fatty acid metabolism’ (e.g., *ACOX1*, *CPT1A*, *NUDT7*) was enriched in the meta-analysis DEG but only 4 of the 8 individual dataset DEG. The pathways ‘The citric acid (tricarboxylic acid [TCA]) cycle and respiratory electron transport’ (e.g., *ACO2, COX5A, COX5B*), ‘Citric acid cycle (TCA cycle)’ (a subset of genes in ‘The citric acid (TCA) cycle and respiratory electron transport’), ‘Respiratory electron transport, ATP synthesis by chemiosmotic coupling, and heat production by uncoupling proteins’ (e.g., *ATP5MC3, ETFA, NDUFA2*), and ‘Respiratory electron transport’ (a subset of genes in ‘Respiratory electron transport, ATP synthesis by chemiosmotic coupling, and heat production by uncoupling proteins’) were all enriched in the down-regulated DEG for the GSE38713, GSE73661, and GSE114527 datasets, but not in the meta-analysis down-regulated DEG. All 5 ‘Transport and modification-related’ pathways were enriched in the meta-analysis down-regulated DEG (‘Glucuronidation’ [e.g., *UGDH, UGP2, UGT1A1*], ‘Phase I – Functionalization of compounds’ [e.g., *ACSS2, ADH1A, CYP2B6*], ‘Phase II – Conjugation of compounds’ [e.g., *GSTM4, NAT2, SLC26A2*], ‘Response to metal ions’ [e.g., *MTE1, MT1F, MT1G*], and ‘SLC-mediated transmembrane transport’ [e.g., *SLC1A1, SLC3A1, SLC4A4*]) and in the down-regulated DEG for GSE73661 and GSE87466 (Supplementary Fig. S11).

## Discussion

Meta-analysis of datasets derived from studies that included mucosal gene expression analysis in patients with UC resulted in the identification of a UC gene expression disease signature consisting of 946 up- and 543 down-regulated DEG. This signature was derived from multiple studies that included patients with active endoscopic disease, and may be useful as a single reference that is generally applicable to a more diverse population of patients with UC and active inflammation than any single dataset. Eight publicly accessible microarray datasets were included to identify the most robustly regulated genes and Reactome pathways in patients with UC compared to non-IBD controls. Combining multiple datasets increased the overall sample size (16.7 and 7.8 times the median UC and non-IBD control sample sizes) and facilitated the identification of significantly up- or down-regulated DEG in meta-analysis despite minimal change in gene expression in some of the individual datasets. Furthermore, the risk of type-1 error associated with the identification of a gene in 1 dataset but not in others was reduced by combining different datasets. We anticipate that this approach will be of considerable value for future evaluation of gene expression levels across these datasets by other investigators. A web-based data-mining tool has been created to facilitate such research (https://premedibd.com/genes.html).

Several interesting findings were observed in this study. Our meta-analysis DEG included 15 that overlap with previously described IBD susceptibility genes and 16 eQTL genes (Table 3). Additionally, the 10 top-ranked most up-regulated genes included *LCN2*, which encodes an inflammation-induced anti-bacterial protein produced by neutrophils^21^, as well as *DUOX2* and *DUOXA2.* DUOX2 is associated with very early onset IBD^22^ and the genes encode the reactive oxygen species (ROS)-generating enzyme dual oxidase and dual oxidase accessory proteins, respectively, which have also been previously reported to be overexpressed in UC^23^. The 10 top-ranked most down-regulated genes included *AQP8*, a gene encoding a small integral membrane protein that regulates water absorption in the absorptive cells of the duodenum, jejunum, and colon^24^ whose expression is reported to be decreased in patients with UC compared to controls^14,25,26^, and *CLDN8*, a gene encoding a tight junction protein previously shown to be down-regulated in both UC and CD^27^.

This study identified common gene and pathway-level similarities despite large variation in the number of DEG and relative magnitude of gene log2-FC between the individual datasets. Surprisingly, no relationship was found between samples size and the number of DEG, suggesting that other factors contribute to the power to detect DEG in these datasets. (Supplementary Fig. S12). Lower rates of gene log2-FC were observed for the GSE53306 dataset (Fig. 2), which also had low enrichment values for ECM-related pathways (Supplementary Fig. S11) compared to the other datasets. This dataset was derived from RNA isolated from FFPE, while the remaining datasets were derived from RNA isolated from whole biopsy samples. The data on FFPE-derived RNA is conflicting, with some investigators reporting lower quality relative to whole biopsy material with subsequent limitations to downstream analysis^28,29^, whereas other groups have reported satisfactory results. Additional studies are required to determine the impact of starting material (e.g., FFPE vs. frozen biopsy-derived RNA) on gene expression analyses.

We observed some unexpected findings within this meta-analysis which warrant discussion. For example, the pathway ‘SLC-mediated transmembrane transport’ was enriched in the down-regulated DEG identified in meta-analysis, despite only being enriched in the down-regulated DEG of 3 individual datasets. This observation provides support for the power of meta-analysis to identify enriched pathways that cannot be identified in most of the individual datasets. Conversely, neither ’Citric acid cycle (TCA cycle)’ nor ‘The citric acid (TCA) cycle and respiratory electron transport’ pathways were enriched in the down-regulated DEG identified in meta-analysis despite enrichment of these pathways in the down-regulated DEG identified in 4 of the individual datasets, suggesting that meta-analysis at the gene level does not merely provide the same information as the strongest enrichment scores for the individual dataset DEG.

The study had some important limitations. Specifically, there were large inconsistencies in the methods and data reporting conventions in the original studies. Furthermore, the studies did not consistently report patient-level data that are crucial to enable the full power of meta-analysis in IBD. Only 1 of 8 datasets included even basic characteristics (age and sex). Common definitions and nomenclature, akin to those used within clinical trials, would help facilitate dataset compilation and harmonization, including information on patient- and disease-related factors such as demographics, sample source and processing methods (intestinal segment, preservation, isolation of RNA), disease duration and extent, concomitant medications, and measures of disease activity (clinical, endoscopic, and histological). Standardized reporting of this information would facilitate an analysis of the effect of clinical and demographic covariates, the comparison of disease subsets across cohorts by meta-analysis, and improve the potential for patient-targeted therapy. Additionally, while different normalization or correction approaches have been proposed^30^, for this analysis, datasets were normalized by using a ‘late-stage’ integration^31^, whereby test statistics were derived for individual datasets before merging using a random effects model. We believe this sufficiently reduced potential batch effects without removing inherent heterogeneity in patient groups or study design, which is a concern when applying any normalization method^32^. Finally, the use of biopsy material, which is heterogenous with regard to cell type, inherently limits the detection of DEG in specific populations of cells. However, analysis and discovery of cell-type specific changes in gene expression in samples from patients with IBD is possible with the application of single cell technology^33^.

Selection of datasets was limited to those that were available as microarrays at GEO. Although this approach excluded the increasing number of available RNA-seq datasets as well as datasets available at other repositories, such as ArrayExpress, it reduced analytical variability. Furthermore, although these methods resulted in a more robust signature, we did not include studies that enrolled pediatric patients, such as PROTECT^34^, which has provided valuable insights into gene signatures for disease severity and response. The study methods, however, will nevertheless have significant value and provide improved statistical power for analysis of patient subtypes, small disease cohorts, or for identification of signatures associated with treatment response (e.g., responders and non-responders) where small sample size may require the combination of multiple datasets.

Other approaches have been used to combine gene expression datasets from patients with IBD. Zhu et al. performed Robust Rank Aggregation on gene lists from 14 publicly available datasets with 100 up-regulated and 50 down-regulated genes and identified 7 enrichment modules that are similar to the pathways identified in this study^11^. Other investigators have used meta- analysis to study gene expression in blood or peripheral blood mononuclear cells^9,10^, or to compare CD and UC^8^, or response to therapy^35^. The current study advances the field with the analysis of a non-redundant set of samples (those represented in multiple datasets were excluded) and methods that both visualize the contribution of individual datasets to the meta-analysis, and facilitate direct comparison of the confidence intervals for log2-FC between datasets. Finally, these data provide a useful reference to evaluate the expression of potential biomarkers or therapeutic targets, pharmacodynamic markers, or molecular surrogates of UC disease activity, and will serve as an important resource to summarize the critical information available in the ever increasing number of publicly available gene expression datasets for UC.

## Supplementary Information

Supplementary Information 1.

Supplementary Information 2.

## Data Availability

The datasets generated during and/or analysed during the current study are available in the Gene Expression Omnibus repository, https://www.ncbi.nlm.nih.gov/geo/.

## Abbreviations

CD: Crohn’s disease
DEG: Differentially expressed genes
ECM: Extracellular matrix
FC: Fold change
FFPE: Formalin-fixed and paraffin-embedded
GEO: Gene Expression Omnibus
IBD: Inflammatory bowel disease
IL: Interleukin
MCS: Mayo Clinic Score
PCA: Principal component analysis
PC1: First principal component
PC2: Second principal component
TCA: Tricarboxylic acid
TNF: Tumor necrosis factor
UC: Ulcerative colitis
