# Supplementary material for: Meta-analysis of gene expression disease signatures in colonic biopsy tissue from patients with ulcerative colitis
Source: Sci Rep. 2021 Sep 14;11:18243. doi: 10.1038/s41598-021-97366-5 (PMC8440637; doi:10.1038/s41598-021-97366-5)
Supplement: Supplementary file 2 — Supplementary Information 2. [file 41598_2021_97366_MOESM2_ESM.docx]

**SUPPLEMENTARY INFORMATION**

**
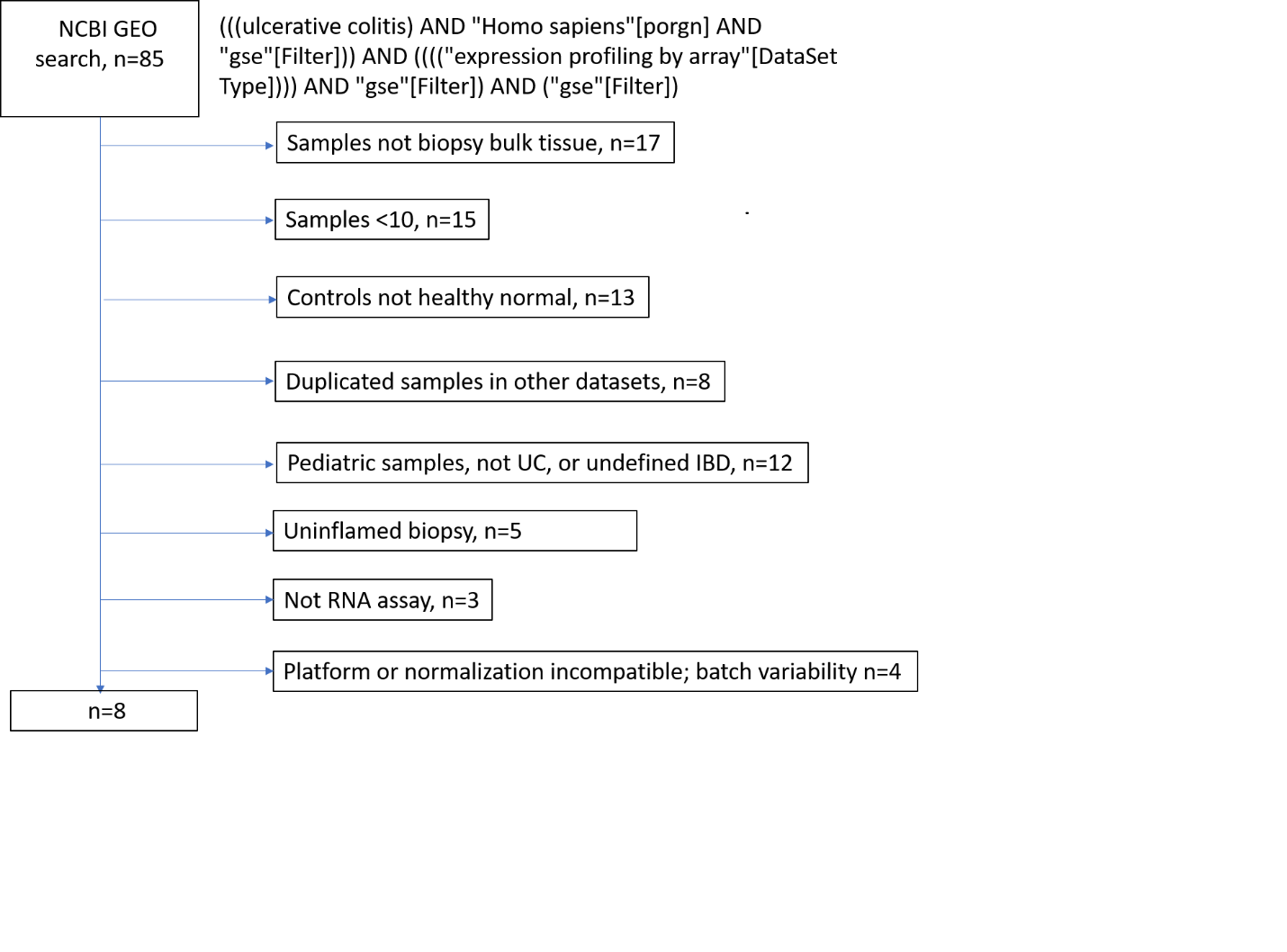
Supplementary Figure S1:** Search criteria and selection of datasets.


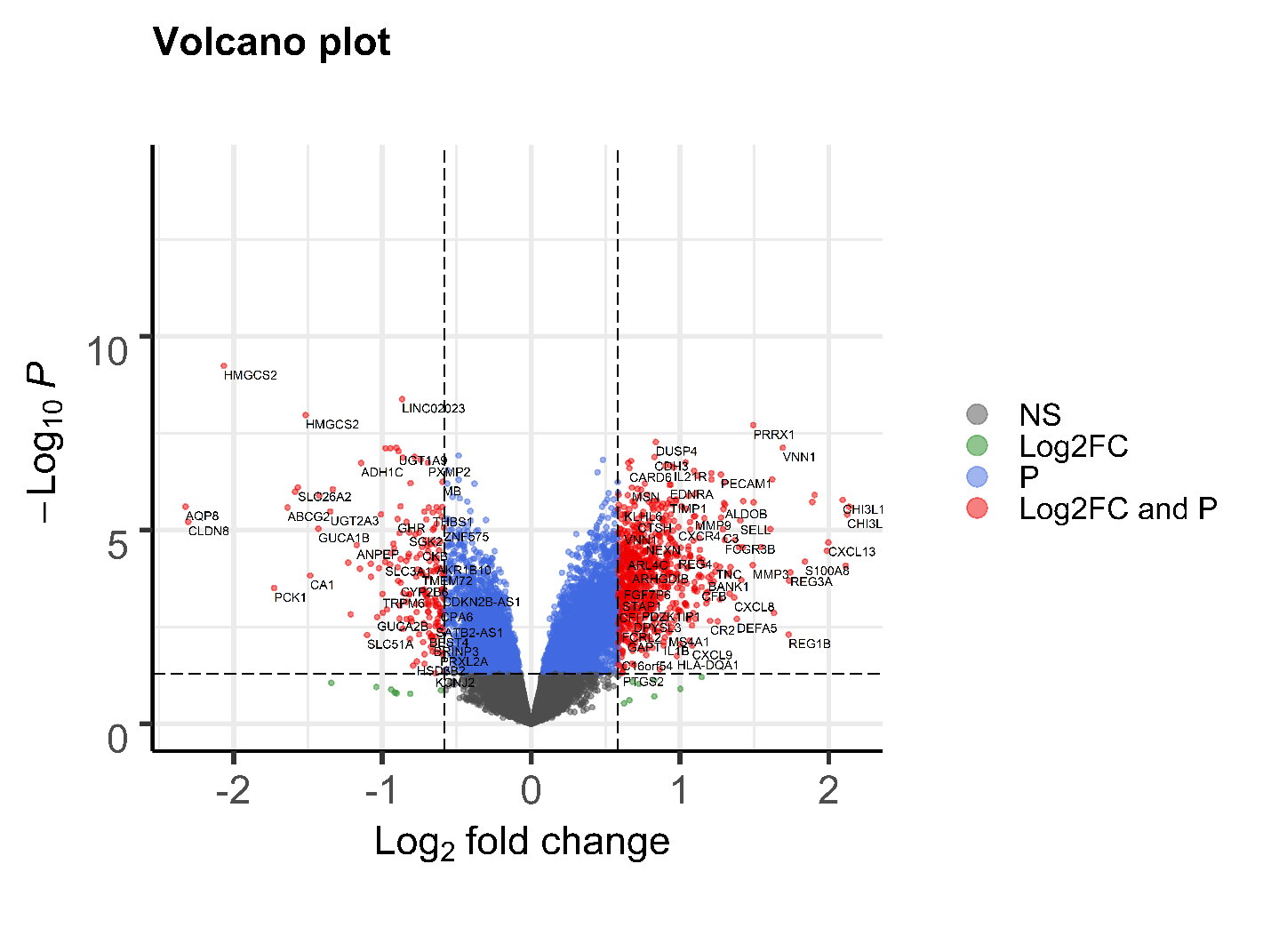


**Supplementary Figure S2**: GSE1337 Volcano plot. Dots to the left of 0 on the X-axis represent genes whose expression is lower in UC patients compared to non-IBD controls, whereas dots to right of 0 on X-axis represent genes whose expression is higher in patients with UC compared to non-IBD controls. Gray dots (NS, not significant) represent genes that do not meet the criteria for log2 fold change (FC) > 1.5 (up or down) or significant adjusted P value <0.05. Green dots represent genes that meet the criterion for log2 FC > 1.5 (up or down) but not adjusted P value < 0.05. Blue dots represent genes with adjusted P value < 0.05 but not log2 FC > 1.5 (up or down). Red dots represent genes that meet both the log2 FC > 1.5 (up or down) and adjusted P value < 0.05 criteria. The horizontal dashed line is located at a value equivalent to the adjusted P value (0.05). Vertical lines are located at + and –1.5 log2 FC.


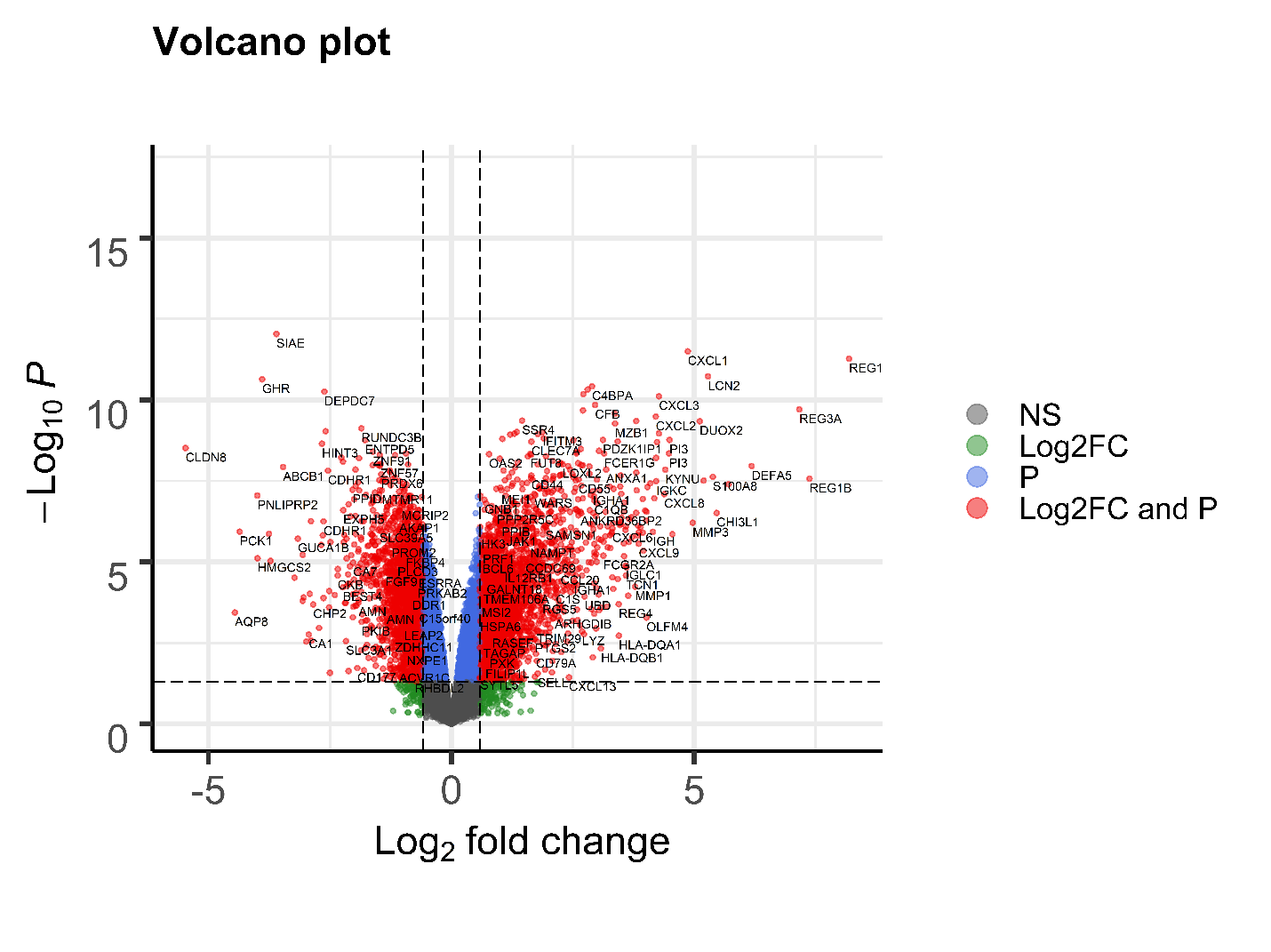
 **Supplementary Figure S3**: GSE9452 Volcano plot. Dots to the left of 0 on the X-axis represent genes whose expression is lower in UC patients compared to non-IBD controls, whereas dots to right of 0 on X-axis represent genes whose expression is higher in patients with UC compared to non-IBD controls. Gray dots (NS, not significant) represent genes that do not meet the criteria for log2 fold change (FC) > 1.5 (up or down) or significant adjusted P value <0.05. Green dots represent genes that meet the criterion for log2 FC > 1.5 (up or down) but not adjusted P value < 0.05. Blue dots represent genes with adjusted P value < 0.05 but not log2 FC > 1.5 (up or down). Red dots represent genes that meet both the log2 FC > 1.5 (up or down) and adjusted P value < 0.05 criteria. The horizontal dashed line is located at a value equivalent to the adjusted P value (0.05). Vertical lines are located at + and –1.5 log2 FC.


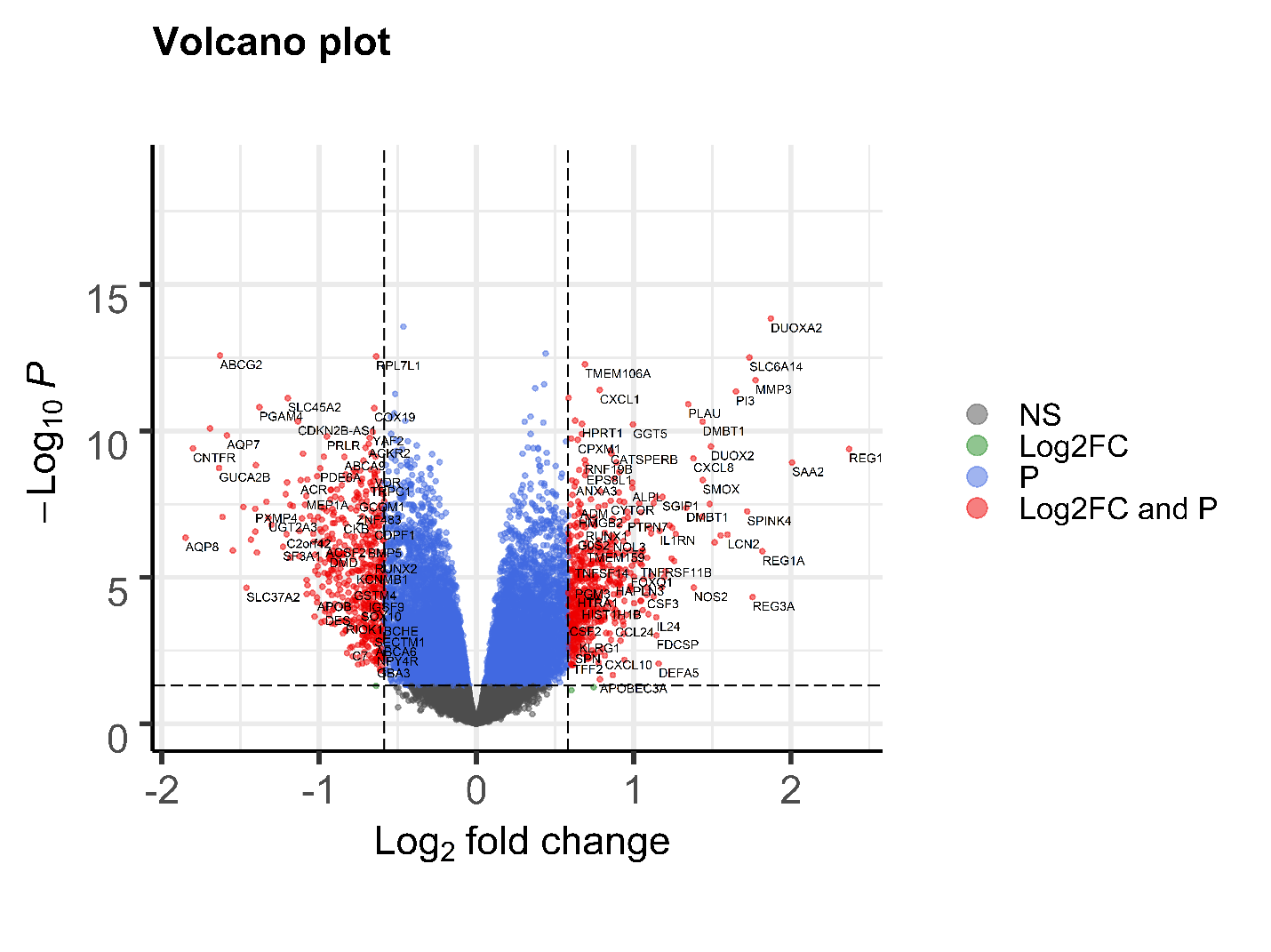


**Supplementary Figure S4**: GSE53306 Volcano plot. Dots to the left of 0 on the X-axis represent genes whose expression is lower in UC patients compared to non-IBD controls, whereas dots to right of 0 on X-axis represent genes whose expression is higher in patients with UC compared to non-IBD controls. Gray dots (NS, not significant) represent genes that do not meet the criteria for log2 fold change (FC) > 1.5 (up or down) or significant adjusted P value <0.05. Green dots represent genes that meet the criterion for log2 FC > 1.5 (up or down) but not adjusted P value < 0.05. Blue dots represent genes with adjusted P value < 0.05 but not log2 FC > 1.5 (up or down). Red dots represent genes that meet both the log2 FC > 1.5 (up or down) and adjusted P value < 0.05 criteria. The horizontal dashed line is located at a value equivalent to the adjusted P value (0.05). Vertical lines are located at + and –1.5 log2 FC.


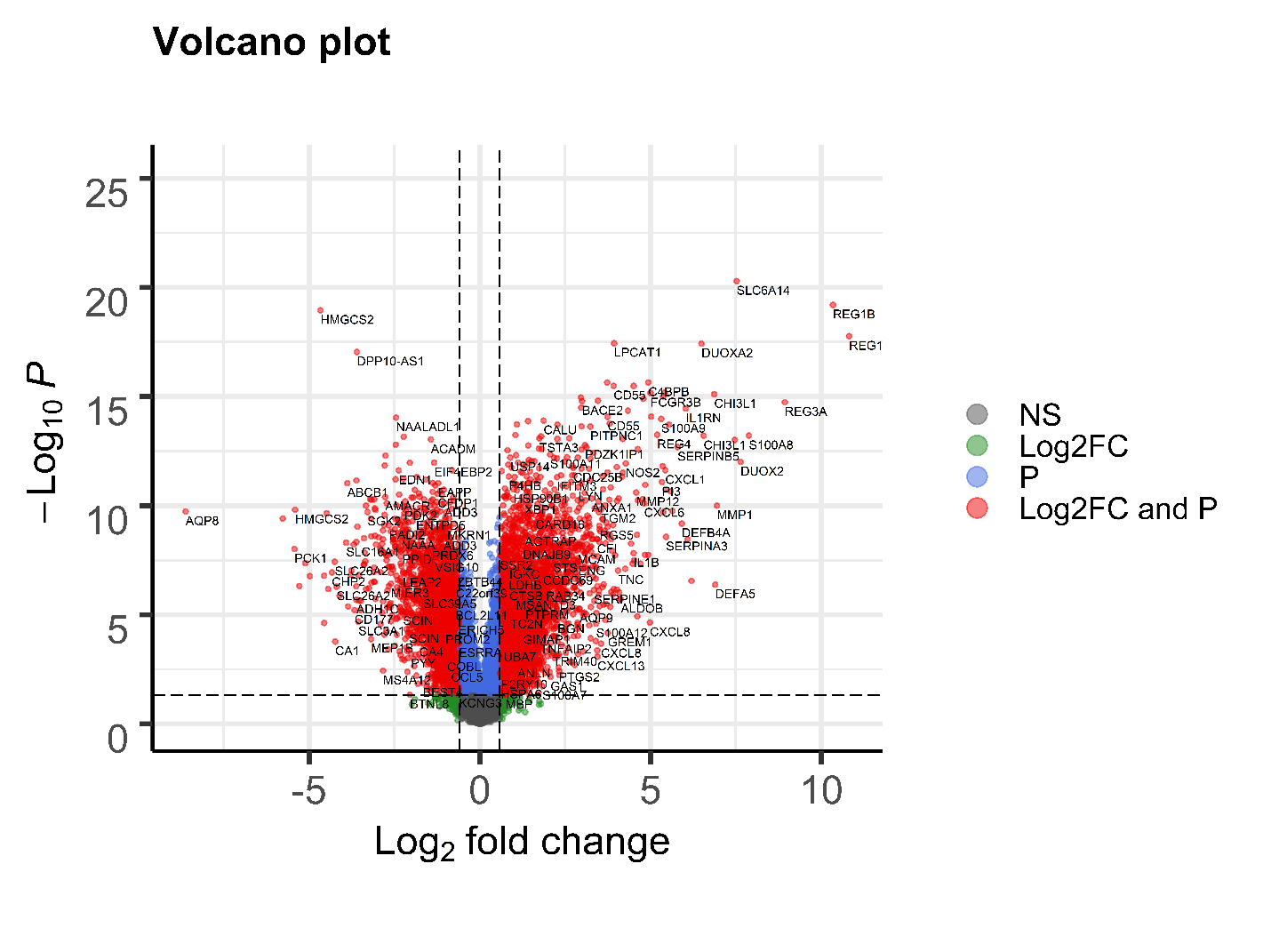


**Supplementary Figure S5**: GSE38713 Volcano plot. Dots to the left of 0 on the X-axis represent genes whose expression is lower in UC patients compared to non-IBD controls, whereas dots to right of 0 on X-axis represent genes whose expression is higher in patients with UC compared to non-IBD controls. Gray dots (NS, not significant) represent genes that do not meet the criteria for log2 fold change (FC) > 1.5 (up or down) or significant adjusted P value <0.05. Green dots represent genes that meet the criterion for log2 FC > 1.5 (up or down) but not adjusted P value < 0.05. Blue dots represent genes with adjusted P value < 0.05 but not log2 FC > 1.5 (up or down). Red dots represent genes that meet both the log2 FC > 1.5 (up or down) and adjusted P value < 0.05 criteria. The horizontal dashed line is located at a value equivalent to the adjusted P value (0.05). Vertical lines are located at + and –1.5 log2 FC.


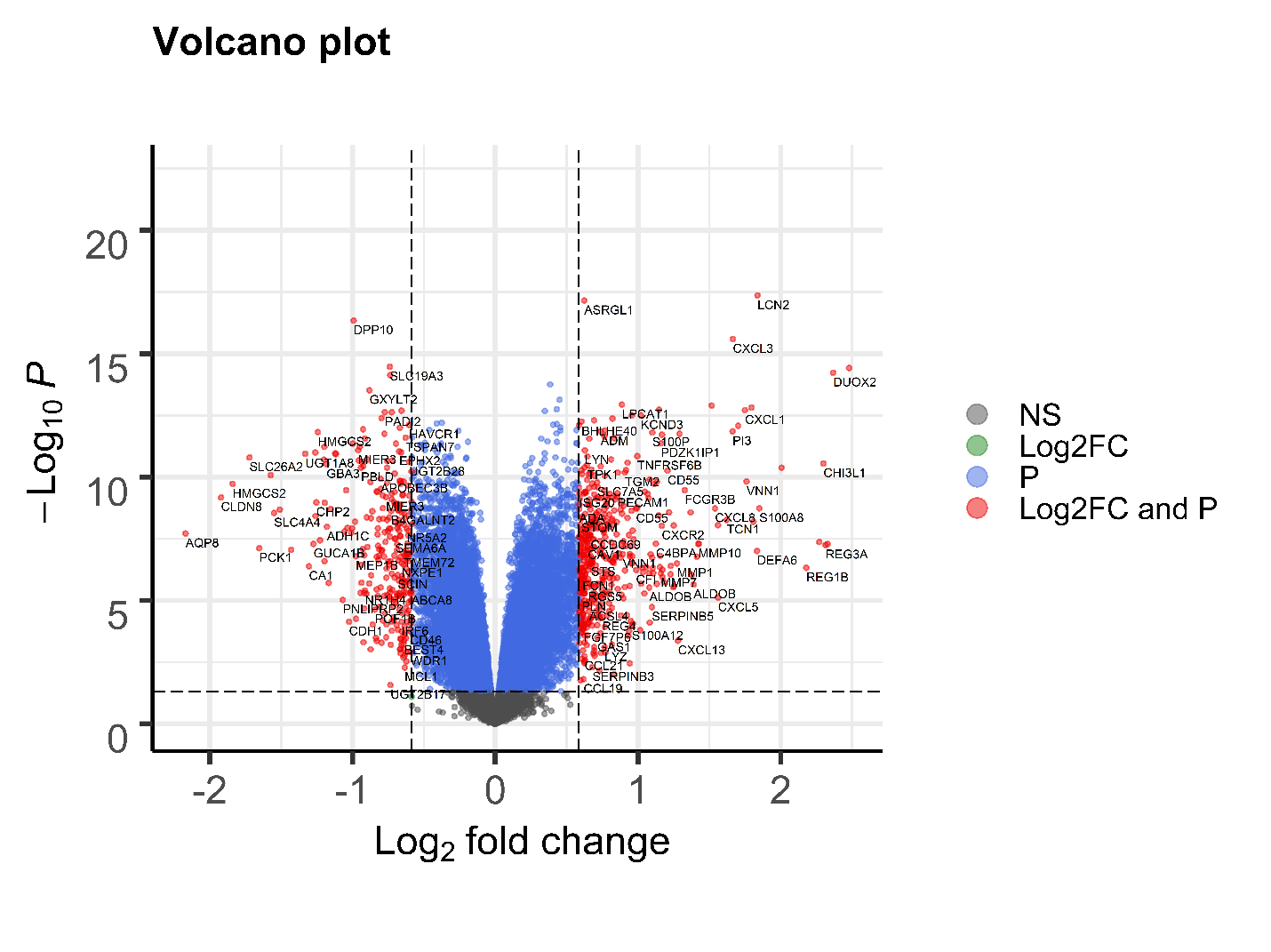


**Supplementary Figure S6**: GSE47908 Volcano plot. Dots to the left of 0 on the X-axis represent genes whose expression is lower in UC patients compared to non-IBD controls, whereas dots to right of 0 on X-axis represent genes whose expression is higher in patients with UC compared to non-IBD controls. Gray dots (NS, not significant) represent genes that do not meet the criteria for log2 fold change (FC) > 1.5 (up or down) or significant adjusted P value <0.05. Green dots represent genes that meet the criterion for log2 FC > 1.5 (up or down) but not adjusted P value < 0.05. Blue dots represent genes with adjusted P value < 0.05 but not log2 FC > 1.5 (up or down). Red dots represent genes that meet both the log2 FC > 1.5 (up or down) and adjusted P value < 0.05 criteria. The horizontal dashed line is located at a value equivalent to the adjusted P value (0.05). Vertical lines are located at + and –1.5 log2 FC.


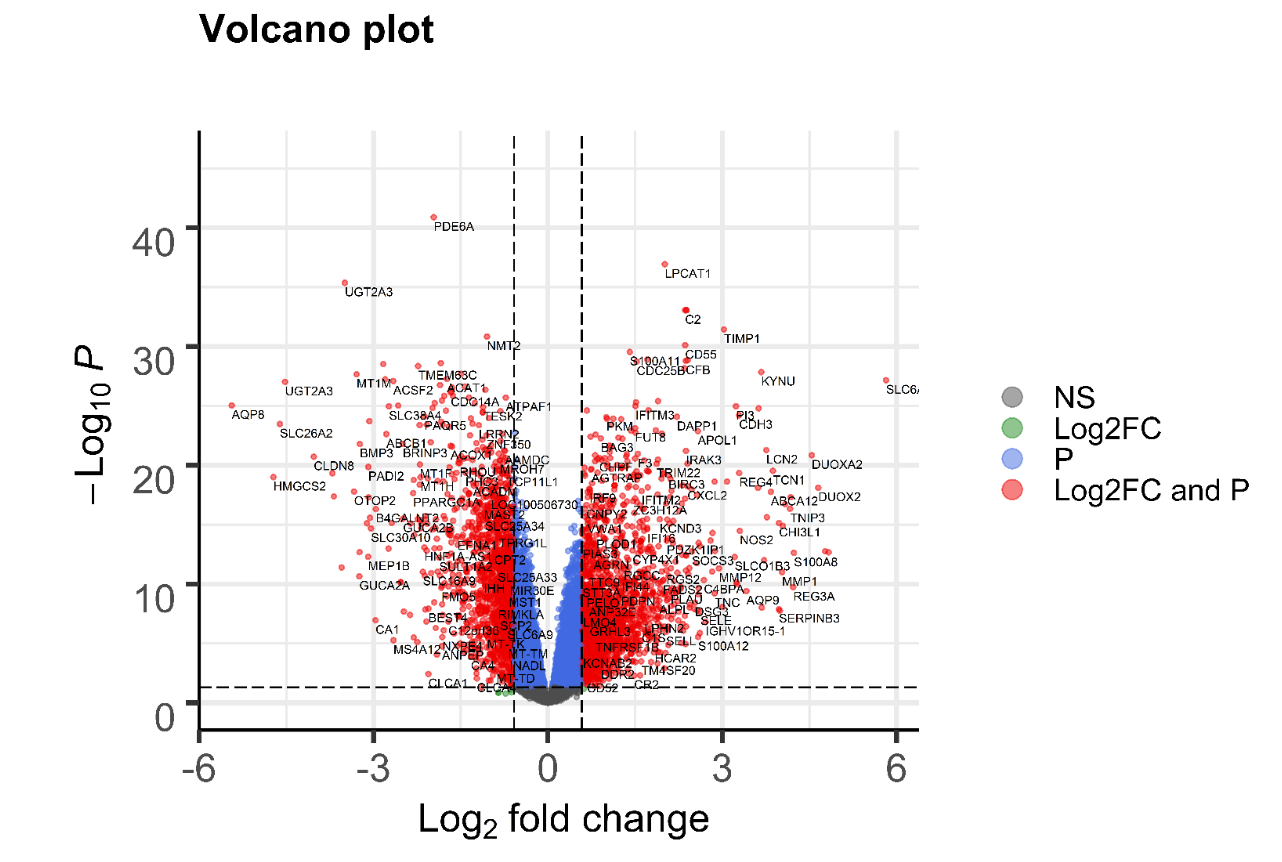


**Supplementary Figure S7**: GSE73661 Volcano plot. Dots to the left of 0 on the X-axis represent genes whose expression is lower in UC patients compared to non-IBD controls, whereas dots to right of 0 on X-axis represent genes whose expression is higher in patients with UC compared to non-IBD controls. Gray dots (NS, not significant) represent genes that do not meet the criteria for log2 fold change (FC) > 1.5 (up or down) or significant adjusted P value <0.05. Green dots represent genes that meet the criterion for log2 FC > 1.5 (up or down) but not adjusted P value < 0.05. Blue dots represent genes with adjusted P value < 0.05 but not log2 FC > 1.5 (up or down). Red dots represent genes that meet both the log2 FC > 1.5 (up or down) and adjusted P value < 0.05 criteria. The horizontal dashed line is located at a value equivalent to the adjusted P value (0.05). Vertical lines are located at + and –1.5 log2 FC.


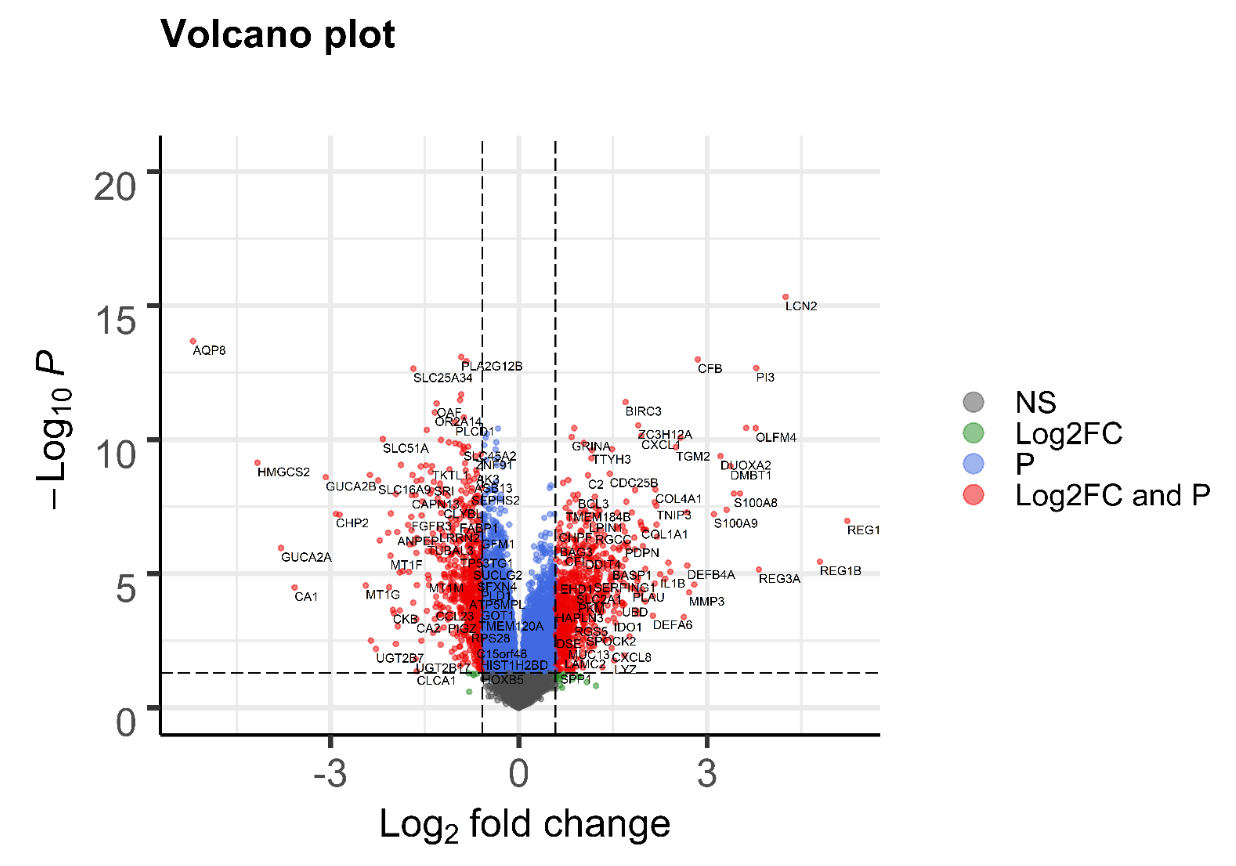


**Supplementary Figure S8**: GSE114527 Volcano plot. Dots to the left of 0 on the X-axis represent genes whose expression is lower in UC patients compared to non-IBD controls, whereas dots to right of 0 on X-axis represent genes whose expression is higher in patients with UC compared to non-IBD controls. Gray dots (NS, not significant) represent genes that do not meet the criteria for log2 fold change (FC) > 1.5 (up or down) or significant adjusted P value <0.05. Green dots represent genes that meet the criterion for log2 FC > 1.5 (up or down) but not adjusted P value < 0.05. Blue dots represent genes with adjusted P value < 0.05 but not log2 FC > 1.5 (up or down). Red dots represent genes that meet both the log2 FC > 1.5 (up or down) and adjusted P value < 0.05 criteria. The horizontal dashed line is located at a value equivalent to the adjusted P value (0.05). Vertical lines are located at + and –1.5 log2 FC.


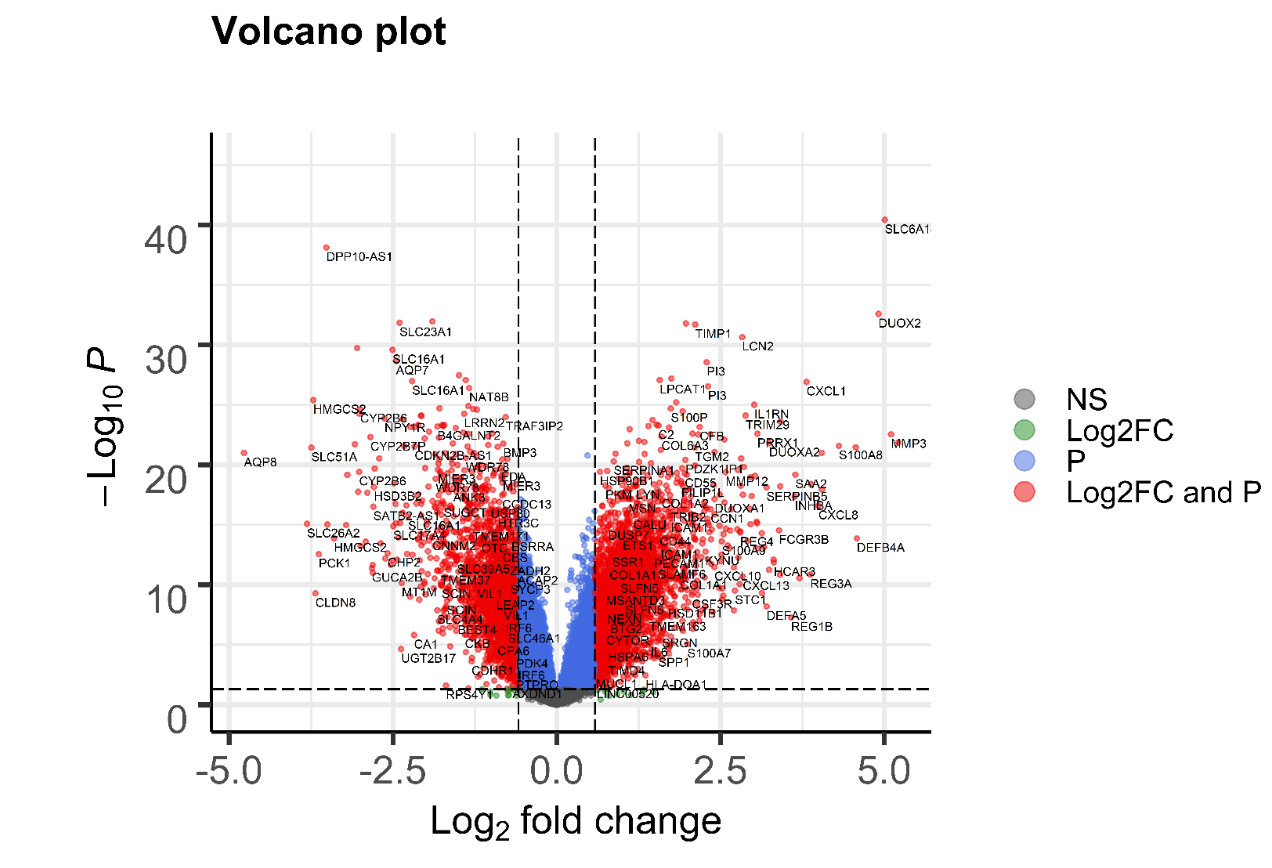
 **Supplementary Figure S9**: GSE87466 Volcano plot. Dots to the left of 0 on the X-axis represent genes whose expression is lower in UC patients compared to non-IBD controls, whereas dots to right of 0 on X-axis represent genes whose expression is higher in patients with UC compared to non-IBD controls. Gray dots (NS, not significant) represent genes that do not meet the criteria for log2 fold change (FC) > 1.5 (up or down) or significant adjusted P value <0.05. Green dots represent genes that meet the criterion for log2 FC > 1.5 (up or down) but not adjusted P value < 0.05. Blue dots represent genes with adjusted P value < 0.05 but not log2 FC > 1.5 (up or down). Red dots represent genes that meet both the log2 FC > 1.5 (up or down) and adjusted P value < 0.05 criteria. The horizontal dashed line is located at a value equivalent to the adjusted P value (0.05). Vertical lines are located at + and –1.5 log2 FC.


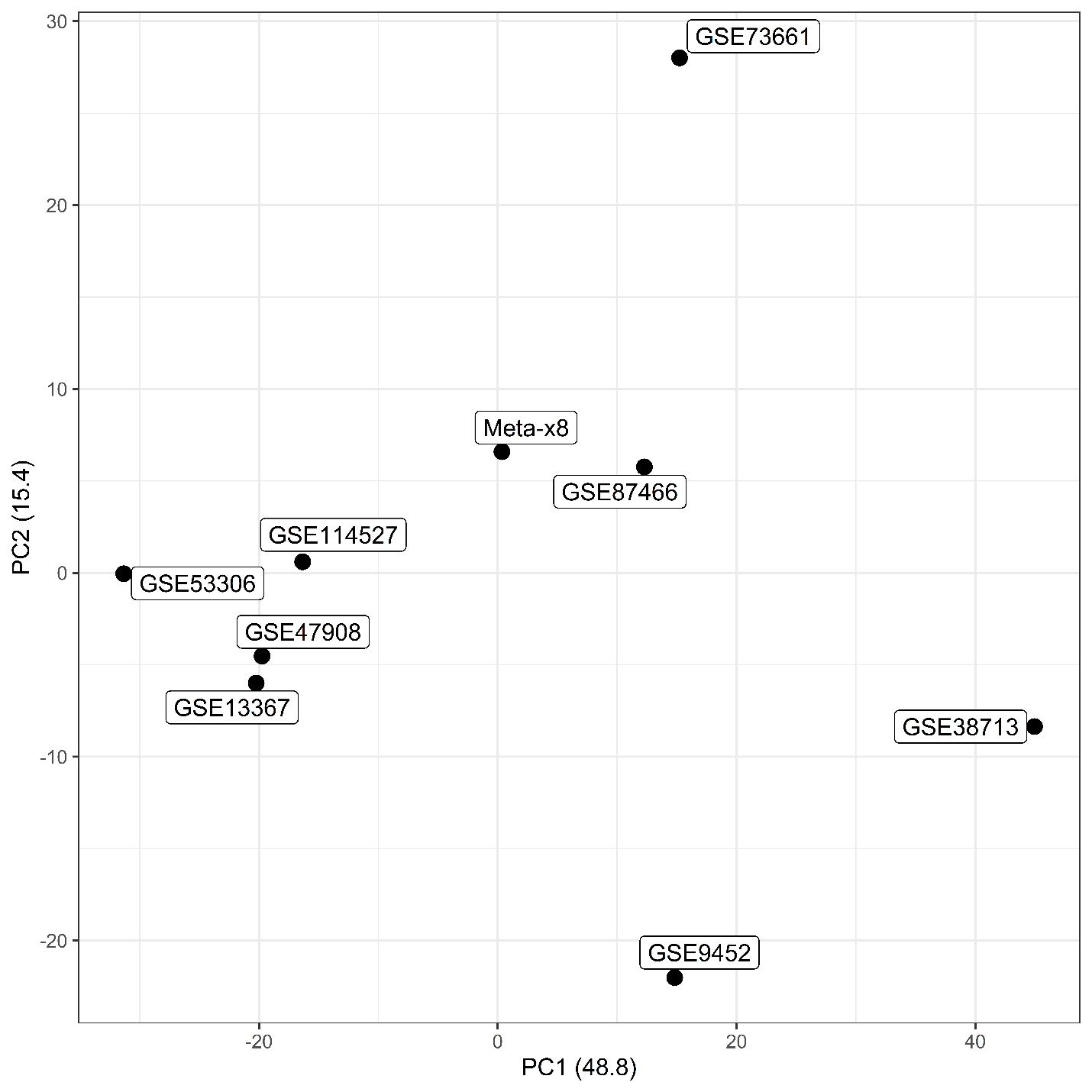


**Supplementary Figure S10**: Principal Component Analysis of datasets by log2 fold change of all genes. PC1, principal component 1. PC2, principal component 2. Values in parentheses indicate percent of variance explained in each PC. Meta-x8 represents the meta-analysis dataset.

A


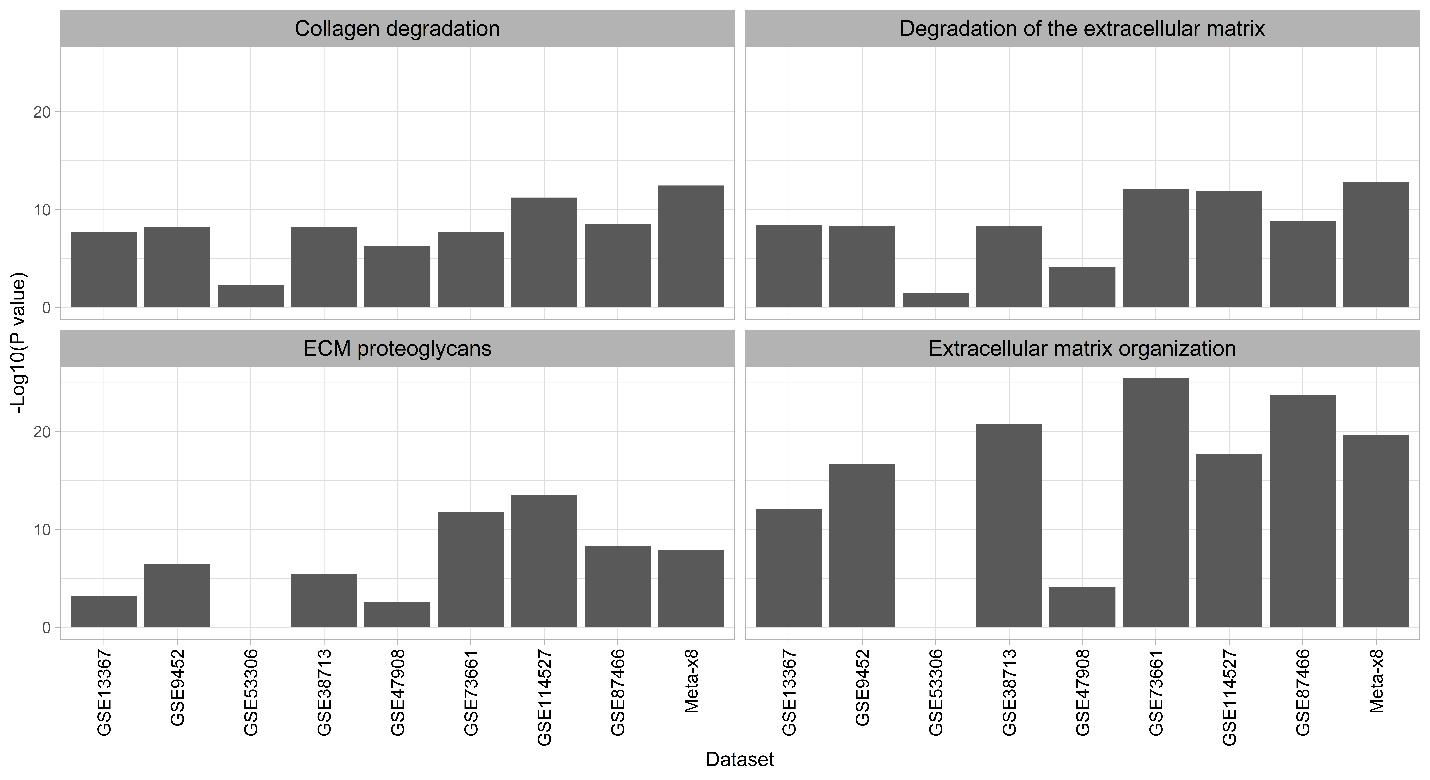


B


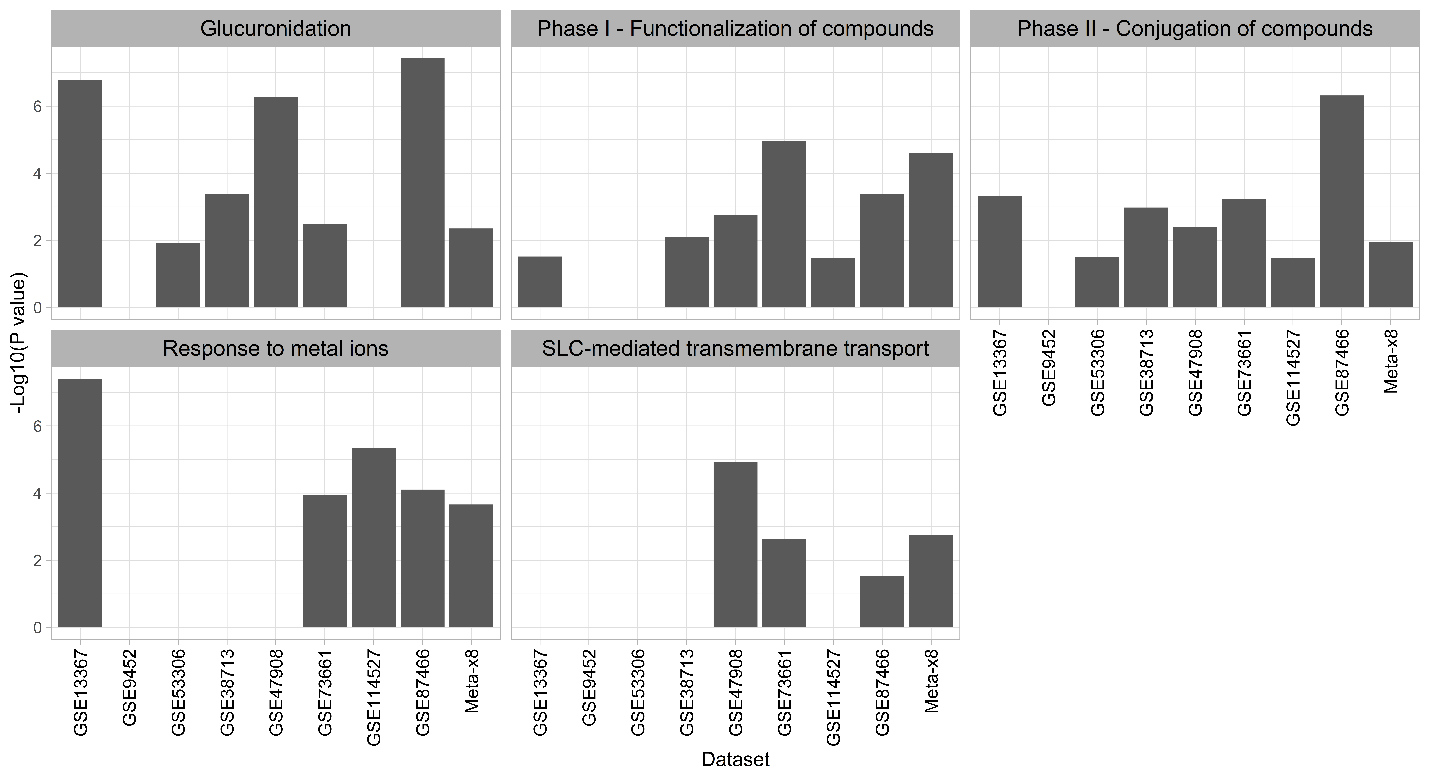


**Supplementary Figure S11**: A, Enrichment of Extracellular Matrix-related Reactome pathways in individual datasets and meta-analysis (Meta-x8) up-regulated genes. B. Enrichment of Transport and modification-related Reactome pathways in individual dataset and meta-analysis (Meta-x8) down-regulated genes. The log10 of the adjusted P value is shown on the Y-axis with higher bars representing lower adjusted P values.

A B


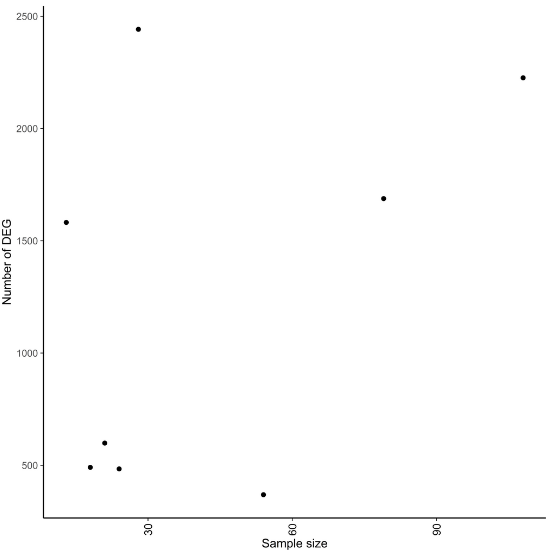


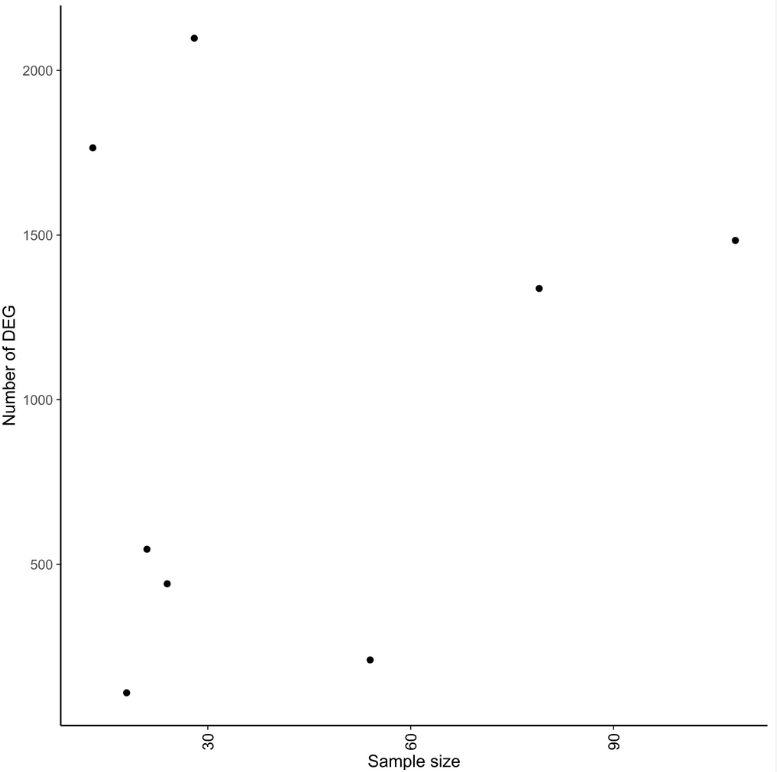


C


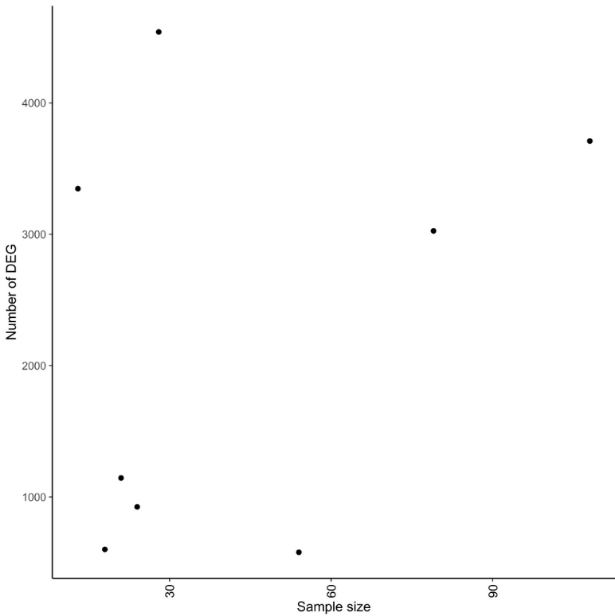


**Supplementary Figure S12**. The association between sample size (total number of total subjects, UC and non-IBD controls) and the number of differentially expressed genes (DEG) in the individual datasets was investigated graphically and by determining Spearman’s correlation coefficient (ρ). A, Up regulated DEG and total samples size (ρ= .21). B, Down regulated DEG and total samples size (ρ= .24), C Up or Down regulated (total) DEG and total samples size (ρ= .31).

**SUPPLEMENTARY METHODS**

Each dataset was downloaded from GEO using GEOquery ^1^ to create an ExpressionSet containing the intensity values, phenotype data (metadata), and gene feature information. Datasets were previously normalized and log2 transformed by the authors.

Probesets in each dataset were annotated in R^2^ with the annotate^3^ package using annotation indicated in the GEO description (<https://www.ncbi.nlm.nih.gov/geo/query/acc.cgi?acc=GSE13367> ; <https://www.ncbi.nlm.nih.gov/geo/query/acc.cgi?acc=GSE9452> ; <https://www.ncbi.nlm.nih.gov/geo/query/acc.cgi?acc=GSE53306> ; <https://www.ncbi.nlm.nih.gov/geo/query/acc.cgi?acc=GSE38713> ; <https://www.ncbi.nlm.nih.gov/geo/query/acc.cgi?acc=GSE47908> ; <https://www.ncbi.nlm.nih.gov/geo/query/acc.cgi?acc=GSE73661> ; <https://www.ncbi.nlm.nih.gov/geo/query/acc.cgi?acc=GSE114527> ; <https://www.ncbi.nlm.nih.gov/geo/query/acc.cgi?acc=GSE87466>). All probesets were retained (i.e., those without gene symbols were not excluded) for all datasets if possible, but for GSE53306 the authors previously had removed 2919 absent genes.

Differential gene expression was determined using limma,^4^ as it is most suited for microarray analysis, and because of its extensive use in this context (for example see ^5,6^) and maturity. The parameter confint was set to T, and significance was defined as a log2 fold change (FC) > log2(1.5) (up or down) and with adjusted P value (Benjamini Hochberg) < .05 when comparing UC to non-IBD control samples. The analysis was not adjusted for covariates, such as age or sex since patient level data was not available for most datasets. Multiple Probesets for the same gene were summarized at the Gene Symbol level (and counted as 1 differentially expressed gene). Visualization of log2-FC and P value was performed using the R package EnhancedVolcano.^7^

Meta-analysis of DEG in the 8 datasets was performed using the R package ‘MetavolcanoR’^8^, using the topTable output of each dataset from limma (including the confidence interval (CI)) and the parameters cvar = T, collaps = T [sic], and metathr = 0.1. An additional Benjamini Hochberg P value correction was calculated for each gene following metanalysis. For meta-analysis, DEG were defined as those regulated in the same direction in 6/8 datasets, an up or down log2‑FC > log2(1.5), and an adjusted P value < .05. The top 10 up- and down-regulated DEG, as ranked by TopConfects approach^9^ were also identified and presented in Table 2.

To reduce dimensions of the data with summary variables for all genes, principle component analysis was conducted on log2-FC values for all genes. Analysis was done using ‘prcomp’ in R base package stats and pcaExplorer.^10^

IBD Susceptibility Genes were obtained from de Lange et al previously published summary statistics from 12,882 IBD cases and 21,770 population controls imputed using the 1000 Genomes Project reference panel.^11^ IBD eQTL were compiled from previous publications: Table 1 from Momozama et al. ^12^ and Table 2 fromKabkchiev et al.^13^. mQTL were extracted from Agliata et al (4 genes described in text) ^14^.

Gene set enrichment analysis was performed on DEG symbols with the Reactome Pathways using R packages ReactomePA^15^ and clusterProfiler ^16^ with the parameters set to pvalueCutoff = .05, qvalueCutoff = .2, minGSSize = 10, and an additional filter for the final gene count in each significant pathway to be greater than 3. The background genes or ‘universe’ was set to all the gene symbols in the individual dataset or meta-dataset, as applicable. Pathway genes listed in text are examples of genes that were differentially expressed in meta-analysis and not necessarily unique to that pathway (many Reactome pathways have overlapping gene sets).

The top 3 most enriched pathways (highest -log10[padj]) for each of the individual datasets were plotted using ggplot2 ^17^ and compared to the enrichment values for the other datasets and meta-analysis. To simplify the visualization, enriched pathways were arbitrarily grouped for up-regulated DEG pathways into similar groups: “Immune-related” (‘Chemokine receptors bind chemokines’, ‘Interleukin-10 signaling’, ‘Interleukin-4 and Interleukin-13 signaling’, ‘Peptide ligand-binding receptors’, ‘Neutrophil degranulation’, and ‘Signaling by interleukins’), and “Extracellular Matrix-related” (‘Collagen degradation’, ‘Degradation of the extracellular matrix’, ‘ECM proteoglycans’, and ‘Extracellular matrix organization’). Enriched pathways for down-regulated DEG pathways included “Metabolism related” (‘Biological oxidations’, ‘Citric acid cycle (TCA cycle)’, ‘Fatty acid metabolism’, ‘Respiratory electron transport’, ‘Respiratory electron transport, ATP synthesis by chemiosmotic coupling, and heat production by uncoupling proteins’, and ‘The citric acid (TCA) cycle and respiratory electron transport’, and “Transport and Modification-related” ( ‘Glucuronidation’, ‘Phase 1-Functionalization of compounds’, ‘Phase II- Conjugation of compounds’, Response to metal ions’, ‘SLC-mediated transmembrane transport’).

**REFERENCES**

1 Davis, S. & Meltzer, P. S. GEOquery: a bridge between the Gene Expression Omnibus (GEO) and BioConductor. *Bioinformatics* **23**, 1846-1847, doi:10.1093/bioinformatics/btm254 (2007).

2 R Core Team. R: A language and environment for statistical computing. R Foundation for Statistical Computing, Vienna, Austria. <https://www.R-project.org/>. (2017).

3 annotate: Annotation for microarrays. <https://rdrr.io/bioc/annotate/> v. 1.62.0. (2019).

4 Ritchie, M. E. *et al.* limma powers differential expression analyses for RNA-sequencing and microarray studies. *Nucleic acids research* **43**, e47, doi:10.1093/nar/gkv007 (2015).

5 Lu, L., Townsend, K. A. & Daigle, B. J., Jr. GEOlimma: differential expression analysis and feature selection using pre-existing microarray data. *BMC Bioinformatics* **22**, 44, doi:10.1186/s12859-020-03932-5 (2021).

6 Xu, G. *et al.* Bioinformatics Analysis of Key Candidate Genes and Pathways in Ulcerative Colitis. *Biol Pharm Bull* **43**, 1760-1766, doi:10.1248/bpb.b20-00488 (2020).

7 EnhancedVolcano: Publication-ready volcano plots with enhanced colouring and labeling. v. 1.2.0 ( <https://github.com/kevinblighe/EnhancedVolcano>, 2019).

8 MetaVolcanoR: gene expression meta-analysis visualization tool v. 1.1.0 (<https://github.com/csbl-usp/MetaVolcanoR>, 2019).

9 Harrison, P. F., Pattison, A. D., Powell, D. R. & Beilharz, T. H. Topconfects: a package for confident effect sizes in differential expression analysis provides a more biologically useful ranked gene list. *Genome Biol* **20**, 67, doi:10.1186/s13059-019-1674-7 (2019).

10 Marini, F. & Binder, H. Development of applications for interactive and reproducible research: a case study. *Genomics and Computational Biology* **S.1.v.3**, e39, doi:10.18547/gcb.2017.vol3.iss1.e39 (2016).

11 de Lange, K. M. *et al.* Genome-wide association study implicates immune activation of multiple integrin genes in inflammatory bowel disease. *Nat Genet* **49**, 256-261, doi:10.1038/ng.3760 (2017).

12 Momozawa, Y. *et al.* IBD risk loci are enriched in multigenic regulatory modules encompassing putative causative genes. *Nat Commun* **9**, 2427, doi:10.1038/s41467-018-04365-8 (2018).

13 Kabakchiev, B. & Silverberg, M. S. Expression Quantitative Trait Loci Analysis Identifies Associations Between Genotype and Gene Expression in Human Intestine. *Gastroenterology* **144**, 1488-1496.e1483, doi:10.1053/j.gastro.2013.03.001 (2013).

14 Agliata, I. *et al.* The DNA methylome of inflammatory bowel disease (IBD) reflects intrinsic and extrinsic factors in intestinal mucosal cells. *Epigenetics* **15**, 1068-1082, doi:10.1080/15592294.2020.1748916 (2020).

15 Yu, G. & He, Q. Y. ReactomePA: an R/Bioconductor package for reactome pathway analysis and visualization. *Mol Biosyst* **12**, 477-479, doi:10.1039/c5mb00663e (2016).

16 Yu, G., Wang, L. G., Han, Y. & He, Q. Y. clusterProfiler: an R package for comparing biological themes among gene clusters. *OMICS* **16**, 284-287, doi:10.1089/omi.2011.0118 (2012).

17 Wickam, H. *ggplot2: Elegant Graphics for Data Analysis*. (Springer-Verlag New York, 2016).
